# The association between TyG index and cardiovascular mortality is modified by antidiabetic or lipid-lowering agent: a prospective cohort study

**DOI:** 10.1186/s12933-025-02620-z

**Published:** 2025-02-07

**Authors:** Changchang Fang, Nanqin Peng, Jiang Cheng, Xiyu Zhang, Wenli Gu, Zicheng Zhu, Xiaoping Yin, Zhiwei Yan, Jing Zhang, Peng Yu, Xiao Liu

**Affiliations:** 1 Department of Endocrinology and Metabolism, the Second Affiliated Hospital, Jiangxi Medical College, Nanchang University, Nanchang, Jiangxi China; 2 Department of Anesthesiology, the Second Affiliated Hospital, Jiangxi Medical College, Nanchang University, Nanchang, Jiangxi China; 3 Cardiology Division, Department of Medicine, Queen Mary Hospital, The University of Hong Kong, Hong Kong, China; 4 Department of Neurology, Affiliated Hospital of Jiujiang University, Jiujiang, China; 5 Jiujiang Clinical Precision Medicine Research Center, Affiliated Hospital of Jiujiang University, Jiujiang, China; 6 Provincial University Key Laboratory of Sport and Health Science, School of Physical Education and Sport Sciences, Fujian Normal University, Fuzhou, Fujian China; 7 Department of Cardiology, Sun Yat-sen Memorial Hospital of Sun Yat-sen University, Guangzhou, Guangdong China; 8 Cardiovascular & Metabolic Disorders Program, Duke-National University of Singapore Medical School, Singapore, Singapore

**Keywords:** Triglyceride-glucose index, Cardiovascular Disease, All-cause mortality, Hypolipidemic agents

## Abstract

**Background:**

The triglyceride-glucose (TyG) index is recognized as an alternative measure of insulin resistance (IR) and has been linked to the risks of cardiovascular disease (CVD) and mortality. This study aimed to evaluate whether the association between the TyG index and CVD mortality is influenced by the use of antidiabetic and hypolipidemic agents, given their potential modifying effects on the TyG index.

**Methods:**

Participants from the National Health and Nutrition Examination Survey (1999–2018) were included in the study. Mortality outcomes were tracked through linkage with National Death Index records until December 31, 2019. Data on the use of antidiabetic and hypolipidemic medications (including prescribed insulin, diabetic pills, and cholesterol-lowering agents) were self-reported by participants.

**Results:**

A total of 5,046 adults (representing 42,753,806 individuals, weighted mean age 61.08 years [SE: 0.24]; 49.35% female) were analyzed. The TyG index was significantly associated with all-cause and CVD mortality, and these associations were modified by the use of antidiabetic and hypolipidemic agents (*p* < 0.01). Significant interactions were observed between the TyG index and the use of these agents for mortality outcomes after full adjustments (p-value for interaction < 0.05). Exposure-effect analysis revealed a U-shaped relationship between TyG index levels and the risks of all-cause and CVD mortality in participants using these agents, while a linear positive relationship was observed in participants not using these agents.

**Conclusions:**

The use of antidiabetic and hypolipidemic agents modify the association between the TyG index and all-cause and CVD mortality. These findings suggest that future studies on the TyG index and its relationship with CVD and mortality should account for the modifying effects of these agents.

**Graphical abstract:**

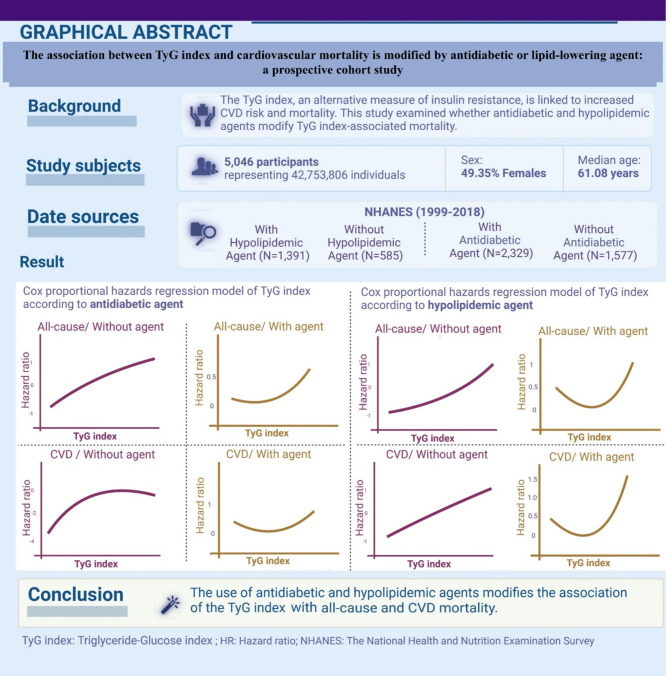

**Supplementary Information:**

The online version contains supplementary material available at 10.1186/s12933-025-02620-z.

## Clinical perspective

### What is already known?

- The TyG index is recognized as a reliable marker for insulin resistance and is associated with increased risks of cardiovascular disease and mortality.

### What is new?

- This nationally representative cohort study showed that the use of antidiabetic or hypolipidemic agents modified the association of the TyG index with all-cause and cardiovascular mortality.
- This study showed a U-shaped relationship between the TyG index and the risks of all-cause and cardiovascular mortality in individuals using antidiabetic or hypolipidemic agents, while a linear positive pattern in those not using these agents.
- The findings highlight the potential modifying effects of antidiabetic and hypolipidemic agents on insulin resistance as measured by the TyG index, suggesting a differential impact on mortality risk depending on agents’ use.

### What are the clinical implications?

- Agents’ usage should be considered in further assessments of TyG associated mortality risk. The complex interplay between these factors suggests that a nuanced approach is essential in the research of TyG index.

## Background

Insulin resistance (IR), defined as reduced sensitivity to the metabolic actions of insulin, is a key pathological feature of type 2 diabetes mellitus (T2DM). IR has been strongly associated with an increased risk of cardiovascular disease (CVD) and mortality [1]. Several methods are currently available for measuring IR, including the hyperinsulinemic-euglycemic (HIEG) clamp test, the homeostasis model assessment of insulin resistance (HOMA-IR), and the homeostasis model assessment of β-cell function (HOMA-β). Among these, the HIEG clamp is considered the gold standard for assessing insulin sensitivity [2]. However, its complexity and high cost limit its use in clinical practice, confining its application primarily to research settings.

The triglyceride-glucose (TyG) index, calculated as ln (fasting triglycerides [mg/dL] × fasting glucose [mg/dL]/2), was first introduced in 2008 by Mendía LE and colleagues [3]. Their findings demonstrated that the TyG index has high sensitivity for identifying insulin resistance in both healthy individuals and the general population [4]. Since both glucose and triglyceride levels are routinely measured in clinical practice, the TyG index has emerged as a simpler and more practical alternative for assessing IR due to its accessibility and affordability. Evidence from previous longitudinal studies, including our own, has shown that the TyG index is associated with an increased risk of various cardiovascular conditions [5–16], including atherosclerosis, myocardial infarction, heart failure, and atrial fibrillation. These findings suggest that the TyG index could be a valuable tool for identifying individuals at high risk for CVD.

However, one of the most critical questions concerns the accuracy of the TyG index. A recent systematic review and meta-analysis reported that the diagnostic accuracy of the TyG index as a surrogate for IR varied [17]. Since the TyG index is directly derived from triglyceride and glucose levels, its accuracy may be influenced by the use of antidiabetic or lipid-lowering medications. For example, basal insulin, often prescribed for diabetes management, affects fasting glycemic control. Similarly, lipid-modifying agents such as fibrates or statins can lead to dynamic fluctuations in triglyceride levels due to the body’s regulatory mechanisms. While statins reduce cholesterol synthesis in the liver, fibrates primarily lower triglyceride levels and improve lipid profiles through different mechanisms. Despite these potential influences, no study to date have examined whether the use of these agents modifies the relationship between the TyG index and cardiovascular outcomes.

This study aimed to evaluate the associations between the TyG index and cardiovascular diseases by stratifying individuals based on their use of antidiabetic and hypolipidemic agents. Considering the potential modifying effects of these agents, we hypothesized that the relationship between the TyG index and CVD risk would differ depending on the use of antidiabetic and lipid-lowering medications.

## Materials and methods

### Data sources and study subjects

The National Health and Nutrition Examination Survey (NHANES) is a research program aimed at evaluating the health and nutritional status of both adults and children in the United States. Conducted by the Centers for Disease Control and Prevention (CDC), the NHANES provides essential health statistics for the nation. The study protocols have undergone thorough review and received approval from the Research Ethics Review Board of the National Center for Health Statistics (NCHS). Informed written consent was obtained from all participants to ensure that their rights were protected. Additionally, to support transparency and further research, the datasets used in this study are publicly available on the official NHANES website (https://www.cdc.gov/nchs/nhanes/index.html).

Participants were required to be aged 18 years or above, with exclusions applied to individuals missing data on the TyG index, mortality status, or the use of both antidiabetic and hypolipidemic agents. The selection process, detailed in Online Figure S1, began with 101,316 individuals from the NHANES 1999–2018 datasets. Exclusions were made for participants under 18 years old (*n* = 42,112), those with incomplete TyG index data (*n* = 33,822), those with missing information on the use of agents (*n* = 19,895), those with missing mortality data (*n* = 6), and those with missing weight coefficient information (*n* = 441). This resulted in a final cohort of 5,046 participants; data on antidiabetic agent use was available for 3,906 participants, whereas data on hypolipidemic agent use were available for 4,832 participants.

### Measurement of agent usage

Participants were identified as insulin users on the basis of their response to the following question: “Taking insulin now”. A “yes” response indicated insulin use. Similarly, oral hypoglycemic agent use was determined by the question “Take diabetic pills to lower blood sugar”, with a “yes” response indicating the use of these agents. If participants reported using either insulin or oral hypoglycemic agents, they were classified as using antidiabetic agents.

Hypolipidemic agent use was determined by the response to the question “Now taking prescribed medicine”, with a “yes” response indicating the use of hypolipidemic agents. Participants using either insulin or hypolipidemic medication were classified as “with agent use”, whereas those using neither were classified as “without agent use”.

### Measurement of the TyG index

The TyG index was computed via the following formula:

$$ TyG\;index = \ln \left[ {\frac{\begin{gathered} fasting\;triglycerides\;(mg/dL) \hfill \\ \times fasting\;glucose\;(mg/dL) \hfill \\ \end{gathered} }{2}} \right] $$

Fasting triglyceride and glucose levels were assessed via enzymatic assays conducted on Roche Modular P and Roche Cobas 6000 chemistry analyzers, respectively. To measure fasting glucose, a hexokinase-mediated reaction was performed on a Roche/Hitachi Cobas C 501 chemistry analyzer [18, 19].

The participants were classified into three groups (T1, T2, and T3) on the basis of the tertiles of the TyG index.

### Assessment of mortality outcomes

To assess patient mortality throughout the follow-up period, we utilized the NHANES Public-Use Linked Mortality Files, which were linked to the National Death Index records up to December 31, 2019. Specific causes of death were identified via the International Statistical Classification of Diseases, 10th Revision (ICD-10). All-cause mortality encompassed deaths from any cause (codes I00–I09, I11–I13, and I20–I51). Cardiovascular deaths were defined as those attributable to rheumatic heart diseases, hypertensive heart and renal diseases, ischemic heart disease, or heart failure (ICD-10 codes I00–I09, I11, I13, and I20–I51). Importantly, revascularization procedures were not included in the definition of cardiovascular events. This study focused solely on cardiovascular mortality as the outcome rather than nonfatal cardiovascular events or procedures [20].

### Measurements of covariates

During household interviews, baseline demographic characteristics were assessed via questionnaires addressing age, sex, race, educational level, smoking status, marital status, drinking status and medication use from NHANES household interviews. Weight and height measurements were obtained at the Mobile Examination Center by trained staff, and the body mass index (BMI) was subsequently calculated via the following formula: weight in kilograms divided by height in meters squared. Race was categorized as Caucasian, African American, Mexican, or other, while education level was classified as less than high school, high school or equivalent, or college or above. The family poverty income ratio (PIR) is used to evaluate socioeconomic status and is calculated as the ratio of a family’s total income to the poverty threshold for a family of the same size and composition.

Systolic and diastolic blood pressures were measured at the Mobile Examination Center, with the final value determined as the average of three consecutive readings. Smoking status was categorized as current smokers, former smokers (participants reported having smoked at least 100 cigarettes in their lifetime but have now quit), and nonsmokers [21, 22]. Drinking status was categorized as current drinkers (those who consumed at least 12 drinks in the past year), former drinkers (those who consumed more than 12 drinks in their lifetime but not in the past year), and nondrinkers [23, 24].

Clinical indicators such as total cholesterol (TC), high-density lipoprotein cholesterol (HDL-C), low-density lipoprotein cholesterol (LDL-C) and fasting triglycerides (TG) were measured at baseline when participants provided blood samples at the Mobile Examination Center.

The Healthy Eating Index (HEI) is a tool for assessing dietary quality, specifically the extent to which a diet al.igns with the Dietary Guidelines for Americans. The HEI-2015 includes 13 components, updated from the HEI-2010. HEI-2015 scores range from 0 to 100, with higher scores indicating better diet quality. For our analysis, we calculated the 13 HEI-2015 components using total nutrient intakes from the first dietary recall day. Physical activity was assessed via the Global Physical Activity Questionnaire developed by the World Health Organization (WHO) [25]. The data were analyzed following the WHO’s analysis guide. Physical activity was expressed in terms of metabolic equivalent (MET) minutes per week for moderate to vigorous physical activity. The respondents were classified on the basis of whether they met or did not meet the recommended physical activity levels: ≥600 MET-minutes per week (equivalent to 150 min of moderate intensity or 75 min of vigorous intensity activity per week) or < 600 MET-minutes per week, in accordance with the physical activity guidelines for adults [26].

Heart failure (HF), coronary heart disease (CHD), heart attack, angina and stroke were defined on the basis of self-reported history. Statin usage includes atorvastatin, simvastatin, rosuvastatin, lovastatin, pravastatin, fluvastatin, and pitavastatin. Hypertension was identified through self-reported history, treatment with antihypertensive medication, or a systolic blood pressure ≥ 130 mm Hg or diastolic blood pressure ≥ 80 mm Hg [27]. Diabetes mellitus (DM) was defined as a fasting plasma glucose level ≥ 126 mg/dL (≥ 7.0 mmol/L) [28], an HbA1c level > 6.5% [29], a diagnosis of DM, or the use of antidiabetic medications (insulin or oral hypoglycemic agents). Dyslipidemia was considered on the basis of self-reported history, treatment with cholesterol-lowering medications, TC ≥ 6.2 mmol/L, or LDL-C ≥ 4.1 mmol/L [30].

### Statistical analysis

The participants were stratified into two distinct baseline TyG index tertiles: (T1 < 8.69, T2 8.69–9.27, T3 ≥ 9.27) and (T1 < 8.59, T2 8.59–9.14, T3 ≥ 9.14). These dataset-specific tertiles were chosen to align with each dataset’s unique distribution, ensuring an even participant distribution across tertiles while minimizing misclassification and underrepresentation within subgroups. Continuous variables are summarized as the means and standard errors (SEs), whereas categorical variables are presented as frequencies and percentages. Baseline characteristics across TyG index tertiles were compared via one-way analysis of variance for continuous variables and Pearson’s chi-square test for categorical variables.

Further analyses included three other insulin resistance indices associated with the TyG index, which were calculated according to the following formula:

1. *HOMA-IR index =* $\frac{{fasting\;glucose\;(mmol/L) \times fasting\;insulin\;(\mu U/mL)}}{{22.5}}$
2. *QUICKI index =* $$\frac{1}{{\log (fasting\;insulin,\mu U/mL)+\log (fasting\;plasma\;glucose,mg/dL)}}$$
3. *HOMA-β index =* $$\frac{{20 \times fasting\;insulin\;(\mu IU/ml)}}{{fasting\;glucose\;(mmol/ml) - 3.5}}$$

Age-, sex-, and race-adjusted linear regression analyses were performed to assess the associations between the TyG index and the insulin resistance indices. Weighted log-rank tests were applied to assess the survival curves according to agent usage and the TyG index. To explore potential non-linear relationships between the TyG index and mortality, we employed restricted cubic spline regression models with three knots (at the 10th, 50th, and 90th percentiles). These analyses were stratified by agent usage to compare the curves and highlight the modifying effects of antidiabetic and hypolipidemic agents on the TyG index. Weighted multivariate Cox proportional hazards regression models were employed to control confounding factors. The crude model was unadjusted; Model I adjusted for age, sex, and race; and Model II further adjusted for BMI, alcohol use status, hypertension, CHD, stroke, angina, and LDL-C. Interaction analyses were performed to evaluate the interaction effects of agent usage and the TyG index on mortality. Additionally, spline models were employed to visualize the interaction, treating the TyG index as a continuous variable after adjusting for age, sex, race, BMI, alcohol use status, hypertension, CHD, stroke, angina, and LDL-C [31].

We performed inverse probability treatment weighting (IPTW) to reduce the baseline imbalance in measured confounders between groups. A standardized mean difference < 0.10 after IPTW indicated no significant between-group difference for the confounder.

IPTW is a statistical method commonly used to reduce confounding in observational studies by balancing baseline covariates across treatment groups. The technique involves calculating propensity scores, which represents the probability of receiving a specific treatment on the basis of observed covariates and assigning weights inversely proportional to these scores. This process generates a pseudo-population where treatment assignment is independent of covariates, effectively mimicking the conditions of a randomized controlled trial [32]. In this study, IPTW was applied to balance baseline characteristics between individuals who did and did not use antidiabetic or hypolipidemic agents. This approach reduces confounding factors and strengthens the validity of causal inferences regarding how these medications modify the relationship between the TyG index and mortality.

In the sensitivity analysis, we incorporated HEI, physical activity, and the PIR as additional confounders in the restricted cubic spline regression models to better understand the modifying effects of antidiabetic and hypolipidemic agents on the association between the TyG index and mortality. We also analyzed the combined effects of both antidiabetic and hypolipidemic agents to explore their joint influence. Additionally, we excluded participants who died within the first two years to minimize the potential for reverse causation.

Stratified analyses were conducted on the basis of sex, age (< 60 years old or ≥ 60 years old), and BMI (< 30.00 or ≥ 30.00).

The statistical analyses were conducted via R software (version 4.2.1; https://www.r-project.org). The R packages “svyjskm” (version 0.5.3), “jskm” (version: 0.5.5) and “ipw” (version 1.0) were used. Sample weights, clustering, and stratification were incorporated into all analyses to account for the complex sampling design of the NHANES, as mandated for NHANES data analysis [33]. *P* < 0.05 was considered statistically significant.

## Result

### Baseline characteristics of study participants

As illustrated in Online Table S1, the cohort comprised 5,046 participants, weighted to represent 42,753,806 individuals (weighted mean age 61.08 years [SE: 0.24]; 49.35% female). The average fasting plasma glucose was 127.44 mg/dL (SE: 0.96), triglyceride was 155.45 mg/dL (SE: 2.40) and TyG index was 8.97 (SE: 0.02). The prevalence of comorbidities was as follows: hypertension (69.92%), diabetes mellitus (65.44%), hyperlipidemia (94.77%), heart failure (7.09%), coronary heart disease (12.93%), and stroke (8.00%). Online Table S2 & S3 shows the individuals’ baseline characteristics stratified by usage of antidiabetic agents or hypolipidemic agents.

### Correlation of TyG index with other insulin resistance indices

**Fig. 1:**
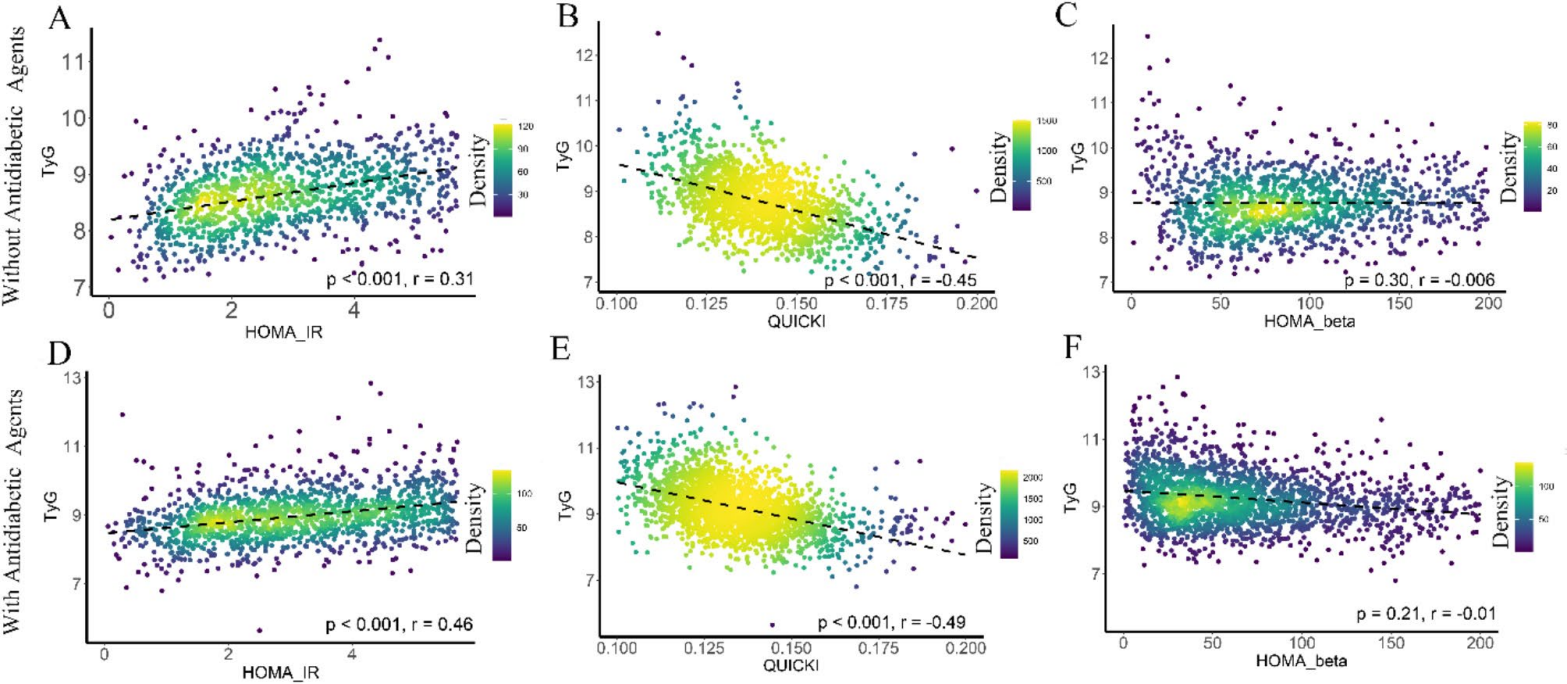
Relationship between the TyG index and other insulin resistance indices (HOMA-IR, QUICKI), and insulin secretory function (HOMA-β), stratified by the use of antidiabetic agents after adjusting for age, sex, and race. Abbreviations: HOMA-β, homeostasis model assessment of β-cell function; HOMA-IR, homeostasis model assessment of insulin resistance; QUICKI, quantitative insulin sensitivity check index

**Fig. 2:**
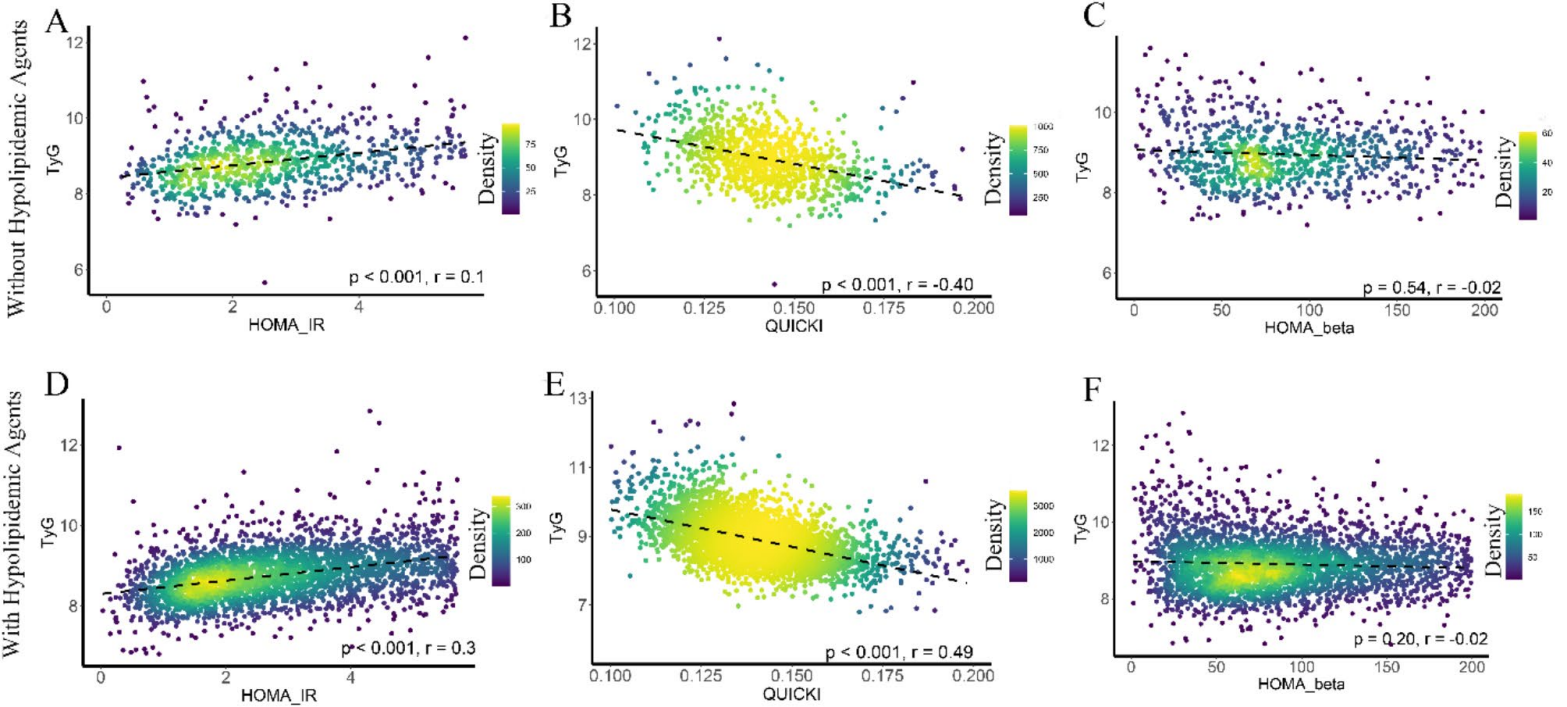
Relationship between the TyG index and other insulin resistance indices (HOMA-IR, QUICKI), and insulin secretory function (HOMA-β), stratified by the use of hypolipidemic agents after adjusting for age, sex, and race. Abbreviations: HOMA-β, homeostasis model assessment of β-cell function; HOMA-IR, homeostasis model assessment of insulin resistance; QUICKI, quantitative insulin sensitivity check index

Figure 1 illustrates the correlations between the TyG index and other insulin resistance indices. In individuals not using antidiabetic agents, the TyG index showed significant correlations with HOMA-IR (*r* = 0.31, *p* < 0.001) and QUICKI (*r* = -0.45, *p* < 0.001), but not with HOMA-β (*r* = -0.006, *p* = 0.30). Similarly, among individuals using antidiabetic agents, the TyG index was significantly correlated with HOMA-IR (*r* = 0.46, *p* < 0.001) and QUICKI (*r* = -0.49, *p* = 0.001), but not with HOMA-β (*r* = -0.001, *p* = 0.21). The correlations between the TyG index and HOMA-IR, QUICKI, and HOMA-β were significantly stronger in individuals using antidiabetic agents compared to those not using these agents (all *p* < 0.05). Consistent findings were observed for individuals using hypolipidemic agents (all *p* < 0.05) and those using both antidiabetic and hypolipidemic agents (all *p* < 0.01), as shown in Fig. 2 and Online Figure S2.

### Associations of TyG index with mortality

#### Exposure-effect analysis

Over a median follow-up period of 9.0 years (interquartile range: 4.5 years), accounting for 6,980 person-years, 827 participants (21.17%) died from any cause, and 246 participants (6.30%) died from cardiovascular causes. The use of antidiabetic or hypolipidemic agents by TyG index tertiles is shown in Online Figures S3 & S4.

The incidence of all-cause and CVD mortality, stratified by the use of antidiabetic or hypolipidemic agents, is presented in Online Figure S5. Participants using antidiabetic or hypolipidemic agents experienced higher rates of all-cause and CVD mortality events (*p* ≤ 0.02).

**Fig. 3:**
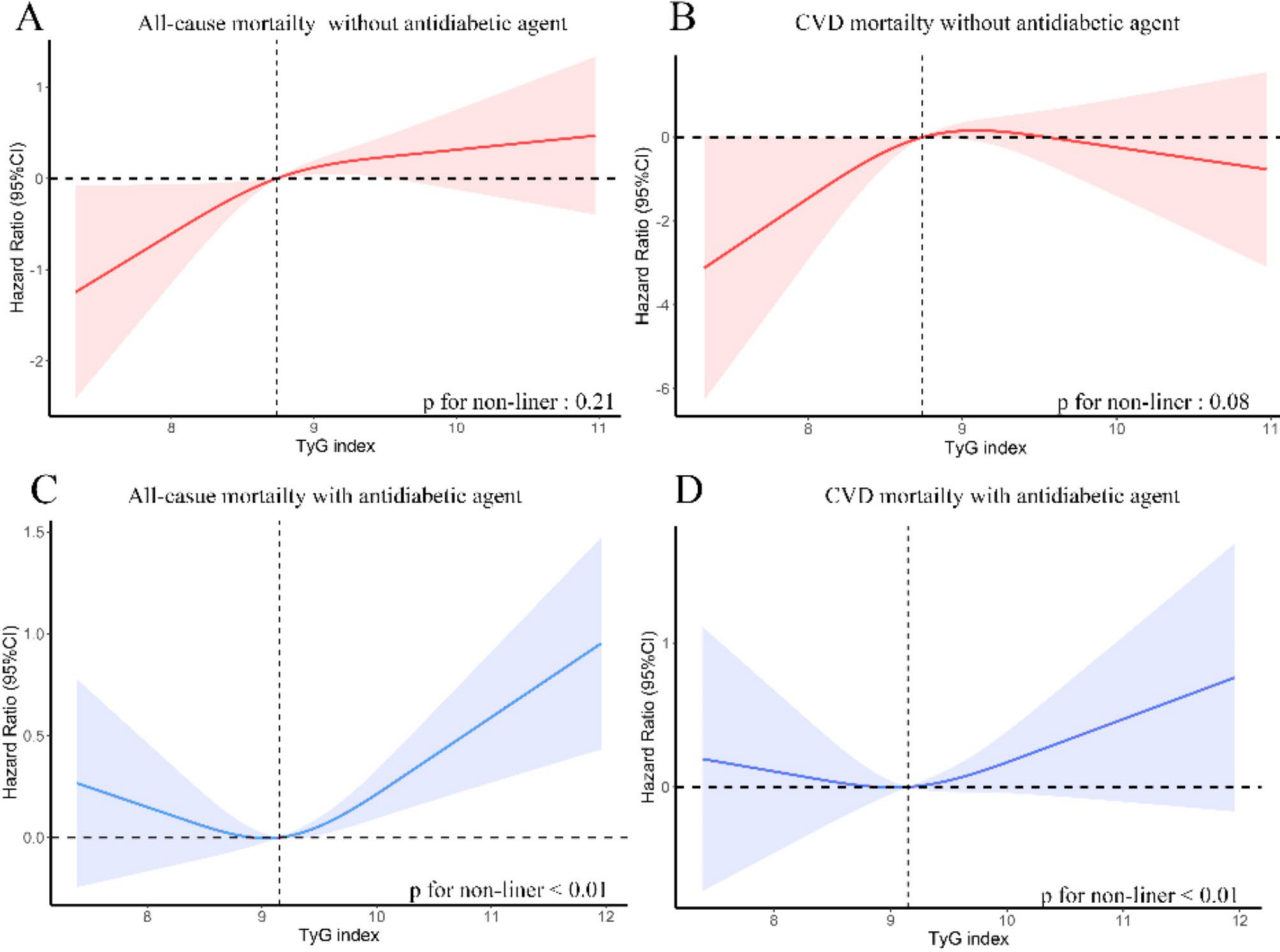
Weighted Cox proportional hazards regression model of TyG index and all-cause (A, C) and CVD mortality (B, D) in general population according to antidiabetic agent after adjusting for age, sex, race, BMI, hypertension status, CHD status, stroke status, angina status, alcohol using status and smoking status. The Hazard ratio of the probability distribution for mortality according to TyG index tertiles. The solid line and red area represent the estimated values and their corresponding 95% CIs, respectively. Abbreviations: BMI: body mass index; CI: Confidence Intervals; CHD: coronary heart disease.; CVD: cardiovascular disease; TyG index: triglyceride-glucose index

Exposure-effect analysis revealed significant differences in the association between the TyG index and all-cause or CVD mortality when stratified by antidiabetic or hypolipidemic agent use. Among participants not using antidiabetic agents, a positive linear relationship was observed between TyG index levels and all-cause mortality (p for non-linearity = 0.21) as well as CVD mortality (p for non-linearity = 0.08) (Fig. 3A and B). In contrast, participants using antidiabetic agents demonstrated a significant U-shaped, non-linear relationship (p for non-linearity < 0.01), with the lowest risks for all-cause and CVD mortality at a TyG index around 9, and increased risks at both lower and higher TyG levels (Fig. 3C and D).

**Fig. 4:**
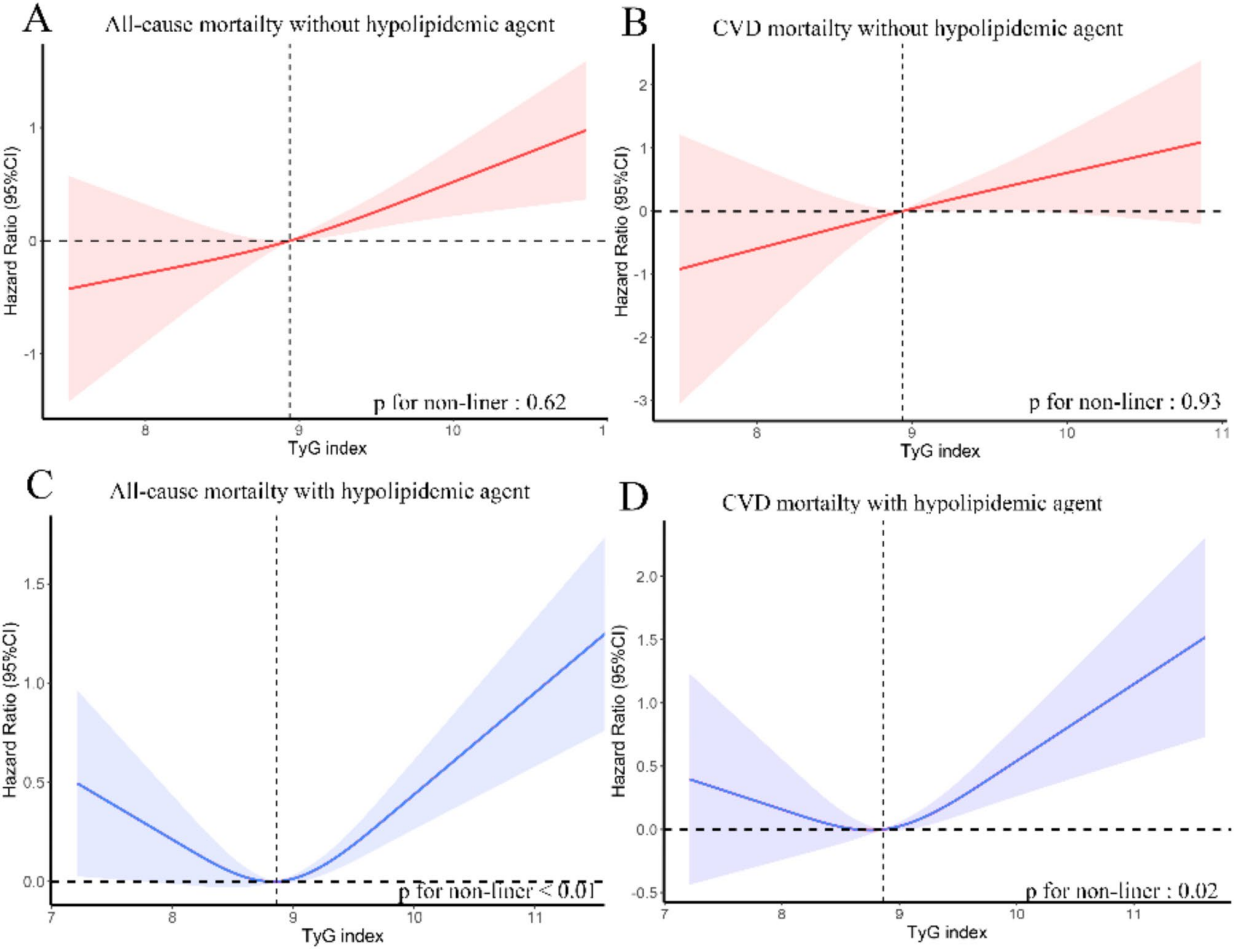
Weighted Cox proportional hazards regression model of TyG index and all-cause (A, C) and CVD mortality (B, D) in general population according to hypolipidemic agent after adjusting for age, sex, race, BMI, hypertension status, CHD status, stroke status, angina status, alcohol using status and smoking status. The hazard ratio of the probability distribution for mortality according to TyG index tertiles. The solid line and area represent the estimated values and their corresponding 95% CIs, respectively. Abbreviations: BMI: body mass index; CI: Confidence Intervals; CHD: coronary heart disease.; CVD: cardiovascular disease; TyG index: triglyceride-glucose index

Similarly, for participants not using hypolipidemic agents, a positive linear relationship was noted between the TyG index and all-cause mortality (p for non-linearity = 0.62) as well as CVD mortality (p for non-linearity = 0.93) (Fig. 4A and B). However, among participants using hypolipidemic agents, a significant U-shaped, non-linear relationship was observed (p for non-linearity ≤ 0.02), with the lowest hazard ratio at a TyG index around 9 and increased mortality risks at both lower and higher levels (Fig. 4C and D).

#### Category and continuous variable analysis

Tables 1 and 2 show the crude and adjusted associations between baseline categorical TyG index and the risks of all-cause and CVD mortality, stratified by agent use. Among participants not using antidiabetic agents, a T2 TyG index (8.69–9.27) was associated with higher all-cause mortality (HR: 1.63, 95% CI: 1.10–2.40, *p* = 0.01) and CVD mortality (HR: 2.56, 95% CI: 1.08–6.07, *p* = 0.03) compared to a T1 TyG index (≤ 8.69) in the fully adjusted model. In participants using antidiabetic agents, a T3 TyG index (≥ 9.27) was significantly associated with increased all-cause mortality compared to a T2 TyG index (HR: 1.32, 95% CI: 1.01–1.71, *p* = 0.04). Significant interactions between the TyG index and antidiabetic agent use were observed for all-cause mortality (p for interaction = 0.002) and CVD mortality (p for interaction = 0.04).

**Table 1:** The associations of TyG index with all-cause and cardiovascular mortality according to the antidiabetic agent usage from the Nation Health and Nutrition Examination Survey 1999–2018

TyG index	Without antidiabetic agent			With antidiabetic agent			*p* for interaction*
	T1 (≤ 8.69)	T2 (8.69–9.27)	T3 (≥ 9.27)	T1 (≤ 8.69)	T2 (8.69–9.27)	T3 (≥ 9.27)	
*All-cause mortality*							0.002
Number of deaths (%)	73 (7.92)	79 (13.53)	61 (18.18)	143 (24.42)	186 (19.79)	284 (26.48)	
Crude Model HR (95%CI)	Ref	1.40 (0.99, 1.97)	1.53 (0.94, 2 49)	1.41 (1.07, 1.86)	Ref	1.23 (0.99, 1.55)	
p value		0.06	0.09	0.01		0.07	
Model I HR (95%CI)	Ref	1.41 (0.99, 2.00)	1.50 (0.97, 2.32)	1.36 (1.07, 1.74)	Ref	1.36 (1.08, 1.71)	
p value		0.06	0.07	0.01		0.01	
Model II HR (95%CI)	Ref	1.63 (1.10, 2.40)	1.61 (0.85, 3.04)	1.14 (0.84, 1.55)	Ref	1.32 (1.01, 1.71)	
p value		0.01	0.15	0.40		0.04	
*CVD mortality*							0.04
Number of deaths (%)	18 (1.78)	21 (3.62)	15 (4.20)	49 (8.12)	54 (5.87)	89 (3.88)	
Crude Model HR (95%CI)	Ref	1.65 (0.88,3.09)	1.59 (0.55, 4.56)	1.58 (0.96, 2.59)	Ref	1.39 (0.91, 2.14)	
p value		0.12	0.39	0.07		0.13	
Model I HR (95%CI)	Ref	1.69 (088,3.26)	1.50 (0.51, 4.40)	1.51 (0.93, 2.45)	Ref	1.54 (1.02, 2.32)	
p value		0.12	0.46	0.10		0.04	
Model II HR (95%CI)	Ref	2.56 (1.08, 6.07)	1.78 (0.59, 5.39)	1.33 (0.72, 2.43)	Ref	1.49 (0.91, 2.45)	
p value		0.03	0.31	0.36		0.11	

Note: Model I: Adjusted for age, sex, race

Model II: Adjusted for age, sex, race, BMI, alcohol using status, hypertension, CHD, stroke, angina, LDL-C

Abbreviations: 95% CI: 95% confidence interval; HR: hazard ratio; Ref: reference; BMI: body mass index; TyG: triglyceride-glucose index; CHD: coronary heart disease

*p for interactions was calculated for Model II

**Table 2:** The associations of tyg index with all-cause and cardiovascular mortality according to the hypolipidemic agent usage from the nation health and nutrition examination survey 1999–2018

TyG index	Without hypolipidemic agent			With hypolipidemic agent			*p* for interaction*
	T1 (≤ 8.59)	T2 (8.59–9.14)	T3 (≥ 9.14)	T1 (≤ 8.59)	T2 (8.59–9.14)	T3 (≥ 9.14)	
*All-cause mortality*							0.03
Number of deaths (%)	42 (6.98)	60 (13.91)	66 (18.38)	274 (16.49)	286 (18.76)	314 (21.86)	
Crude Model HR (95%CI)	Ref	1.69 (0.97, 2.93)	2.17 (1.32, 3.57)	1.48 (0.96, 2.28)	Ref	1.13 (0.78, 1.65)	
p value		0.06	0.002	0.07		0.51	
Model I HR (95%CI)	Ref	1.78 (1.08, 2.93)	2.69 (1.64, 4.44)	1.53 (1.04, 2.26)	Ref	1.36 (0.94, 1.98)	
p value		0.02	< 0.001	0.03		0.10	
Model II HR (95%CI)	Ref	1.58 (0.93, 2.68)	2.19 (1.28, 3.75)	1.41 (1.12, 2 23)	Ref	1.20 (0.79, 1.83)	
p value		0.09	0.004	0.04		0.40	
*CVD mortality*							0.04
Number of deaths (%)	10 (1.66)	18 (3.97)	14 (4.60)	89 (5.42)	84 (5.30)	103 (7.95)	
Crude Model HR (95%CI)	Ref	2.08 (0.79, 5.50)	2.25 (0.84, 6.01)	1.15(0.50, 2.67)	Ref	1.02 (0.55, 1.88)	
p value		0.14	0.10	0.74		0.95	
Model I HR (95%CI)	Ref	2.28 (0.86, 6.02)	3.04 (1.16, 7.94)	1.19 (0.54, 2.60)	Ref	1.19 (0.54, 2.60)	
p value		0.10	0.02	0.67		0.51	
Model II HR (95%CI)	Ref	1.63 (0.54, 4.89)	2.71 (1.88, 8.32)	(0.39, 2.54)	Ref	1.04 (0.55, 1.99)	
p value		0.38	0.04	0.99		0.07	

Note: Model I: Adjusted for Age, sex, race

Model II: Adjusted for age, sex, race, BMI, alcohol using status, hypertension, CHD, stroke, angina, LDL-C

Abbreviations: 95% CI: 95% confidence interval; HR: hazard ratio; Ref: reference; BMI: body mass index; TyG: triglyceride-glucose index; CHD: coronary heart disease

*p for interactions was calculated for Model II

**Fig. 5:**
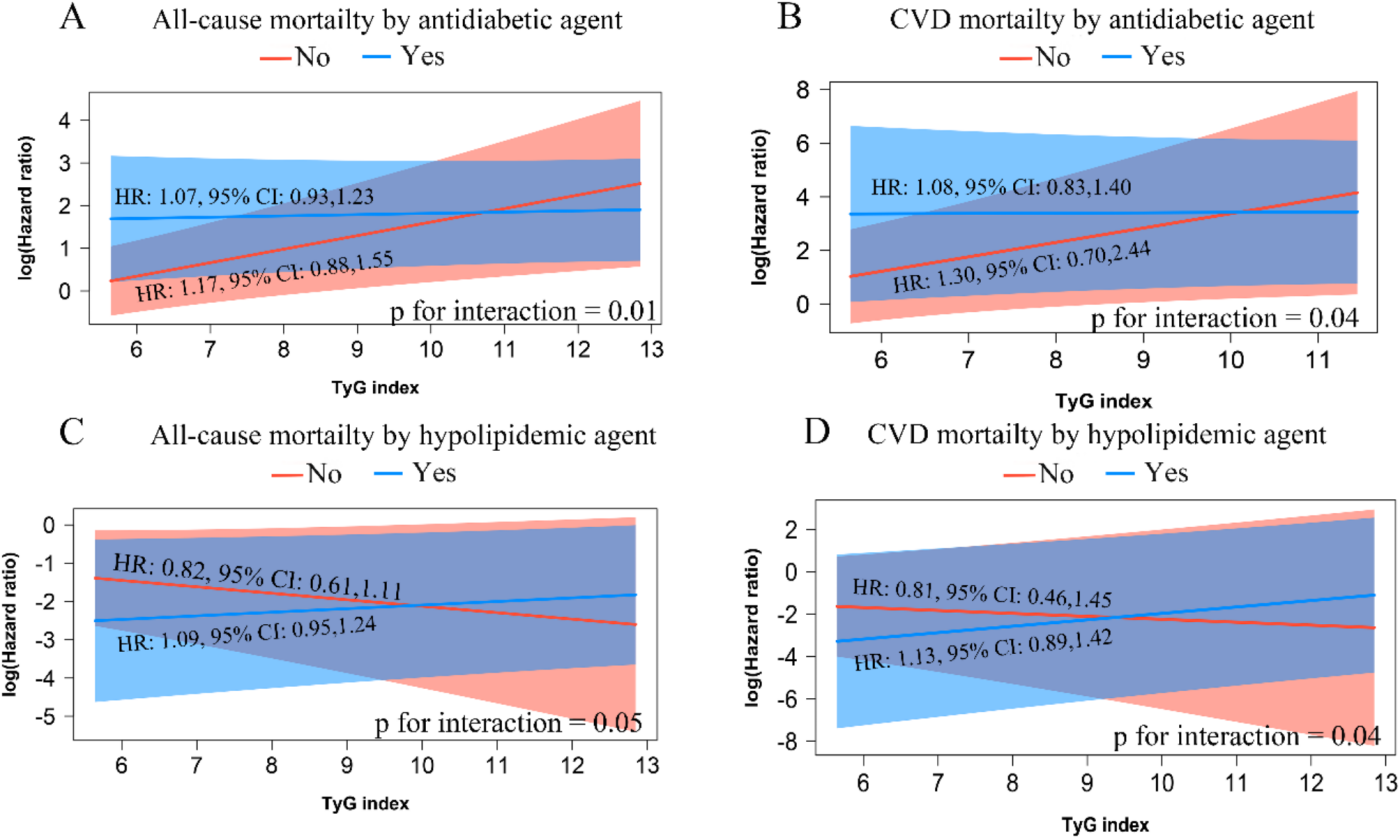
The interaction between TyG index and antidiabetic and hypolipidemic agent usage is associated with all-cause (A, C) and CVD mortality (B, D), adjusting for age, sex, race, BMI, alcohol using status, hypertension, CHD, stroke, angina, LDL-C. Spline models depict the relationship between agent usage and mortality outcomes, stratified by baseline TyG index tertiles. Error bars indicate the 95% confidence intervals. Abbreviations: CHD: coronary heart disease; CI: Confidence Intervals; CVD: cardiovascular disease; TyG index: triglyceride-glucose index

Similar patterns were identified in participants with and without hypolipidemic agents. Significant interactions were observed for all-cause mortality (p for interaction = 0.03) and CVD mortality (p for interaction = 0.04). Figure 5 illustrates the interactions between the TyG index (as a continuous variable) and agent use. Minimal overlap in the interaction lines highlights significant effects on mortality outcomes. Notably, the interactions between the TyG index and antidiabetic agent use were significant for all-cause mortality (p for interaction = 0.01) and CVD mortality (p for interaction = 0.04). Similar interaction patterns were found for participants using hypolipidemic agents, with significant effects on all-cause mortality (p for interaction = 0.05) and CVD mortality (p for interaction = 0.04).

### Sensitivity analysis

The significant interactions between all-cause and CVD mortality and the use of antidiabetic or hypolipidemic agents remained consistent when IPTW was applied (Online Tables S4 & S5). Among participants not using either agent, the TyG index was associated with all-cause mortality (p for non-linearity = 0.02) and CVD mortality (p for non-linearity = 0.67). In contrast, in participants using both agents, a significant U-shaped, non-linear relationship was observed (p for non-linearity < 0.01) (Online Figure S6). Excluding the population that died within 2 years of follow-up yielded results consistent with the main analysis (Online Figure S7). Incorporating the HEI, physical activity, and poverty-income ratio (PIR) as additional confounders also produced consistent dose-response findings (Online Figures S8 & S9).

### Stratified analyses

In participants using antidiabetic agents, a U-shaped relationship was observed between TyG index levels and the risks of both all-cause and CVD mortality, differing from the linear relationship seen in participants not using antidiabetic agents. Similar patterns were observed in participants with and without hypolipidemic agents. However, no significant interaction between baseline TyG index and other stratified variables was identified, as shown in Online Tables S6 & S7.

## Discussion

### Major findings

This nationally representative prospective study examined the interactions between the TyG index and the use of antidiabetic or hypolipidemic agents in relation to all-cause and CVD mortality in the general population. The key finding was the identification of a U-shaped, dose-response relationship between the TyG index and mortality among participants using these agents, while a linear positive association was observed in those not using these agents. Sensitivity analyses using IPTW and combined effects of both agents provided consistent results. These findings offer new insights into how antidiabetic and hypolipidemic agents modify the relationship between the TyG index and cardiovascular risk and mortality.

### Comparisons with previous studies

Previous studies have established a link between the TyG index and an increased risk of CVD [9, 11–14, 34, 35]. However, the shape of the exposure-effect association has varied. For instance, a U-shaped association was reported for new-onset atrial fibrillation in individuals without known CVD [6]. Similarly, some cohorts demonstrated a U-shaped association for CVD in the general population [36–38]. In contrast, other studies reported a positive linear relationship without a notable inflection point [39–42]. These discrepancies may arise from differences in participants’ baseline characteristics, though no definitive explanation for the inconsistency has been identified.

This study demonstrated that the relationship between the TyG index and cardiovascular and all-cause mortality is significantly modified by the use of antidiabetic or hypolipidemic agents. Specifically, a U-shaped association was observed in individuals using these agents, while a positive linear association was seen in those not using them (Figs. 4 and 5). We hypothesize that these medications may modify the observed U-shaped relationship.

Consistent findings were observed in the ChinaPURE Study, a prospective cohort study involving 3 million participants. The study reported a positive linear association between the TyG index and CVD mortality in patients with diabetes but found a significant U-shaped association in those without diabetes [43]. Additionally, evidence from multiple cohorts focusing on patients with diabetes [37, 43, 44] and non-diabetes [39, 45–47] confirmed this distinct dose-response pattern. The above findings support the hypothesis that antidiabetic and hypolipidemic agents can modify the relationship between the TyG index and health outcomes. Other studies have also highlighted the non-linear association between the TyG index and both all-cause and cardiovascular mortality. For example, a population-based cohort study demonstrated that in patients with diabetes or prediabetes using insulin or other antidiabetic medications, the TyG index exhibited a non-linear relationship with mortality, underscoring the importance of considering medication effects when interpreting these associations [9, 40, 48].

Several important confounders should also be considered. In the present study, individuals not receiving treatment were younger compared to those using antidiabetic or hypolipidemic agents. Survival analysis revealed a higher burden of mortality and comorbidities among those on these agents (Fig. 3). These factors may partly explain the distinct associations between the TyG index and mortality stratified by agent use. Consistently, a recent cohort study found that age significantly modifies the relationship between the TyG index and both cardiovascular and all-cause mortality. In that study, a U-shaped association was observed in patients aged over 65 years with T2DM, whereas a positive linear relationship was found in those under 65 years [49]. This may be attributed to the increased susceptibility of older individuals or those with significant comorbidities to hypoglycemia, which can raise the risk of cardiovascular events and mortality. However, in the present study, sensitivity analyses using IPTW, which adjusted for age and other confounders, confirmed the interaction between the TyG index and mortality. Therefore, age differences alone may not fully account for the observed findings. Further studies with age-balanced populations are needed to better understand the role of age in this relationship.

How generalizable are the findings of this study? Although this was a nationally representative prospective study, not all participants had complete records of medication use. Missing data on antidiabetic or hypolipidemic agents in 19.6% of participants could introduce selection bias. Additionally, the accuracy of the TyG index in diagnosing IR remains uncertain. While some studies have demonstrated a moderate diagnostic ability of the TyG index for IR, these studies generally involve small sample sizes and low-to-moderate evidence quality, as highlighted in a recent meta-analysis [50]. Furthermore, the validity of the TyG index has not been fully established in American populations. Additional research is needed to determine whether the observed association between a lower TyG index and higher mortality risk is attributable to IR. Notably, the average BMI in this study population was approximately 30 kg/m², suggesting a higher baseline IR. Therefore, the interaction between the TyG index and the use of antidiabetic or hypolipidemic agents should be further explored in future studies.

### Underlying mechanism

Antidiabetic agents may influence the relationship between the TyG index and mortality through several mechanisms. Insulin reduces blood glucose levels and affects lipid metabolism by suppressing lipolysis and promoting lipogenesis in the liver and adipose tissue. This may lead to lower triglyceride levels and, consequently, influence the TyG index [51].

Hypolipidemic agents, particularly statins and other lipid-lowering drugs, directly reduce triglyceride levels, which are a key component of the TyG index. Some hypolipidemic agents, such as glitazones, improve insulin sensitivity, potentially modifying the association between the TyG index and mortality.

The observed U-shaped—or more precisely V-shaped—relationship can be partially explained. The decrease in mortality with increasing TyG index on the left side of the curve, particularly in individuals using these agents, may be related to risks associated with hypoglycemia. Hypoglycemia has been shown to increase counter-regulatory hormones like adrenaline, which can elevate the risk of cardiovascular and cerebrovascular events, including stroke [52]. Additionally, a prospective cohort study identified low triglyceride levels as a risk factor for hemorrhagic stroke, particularly in women [53]. Low triglyceride levels have also been recognized as predictors of cardiac death in patients [54]. From the TyG index formula, a low TyG index may result from either low triglyceride or low glucose levels. The association between a low TyG index and increased mortality risk may be partially explained by low fasting glucose. A recent systematic review and meta-analysis found that low fasting plasma glucose (< 4.0 mmol/L) is associated with a higher risk of all-cause mortality, major cardiovascular events, and both ischemic and hemorrhagic strokes in individuals without pre-existing cardiovascular disease or diabetes [9]. Other studies have also demonstrated that the relationship between the TyG index and both cardiovascular and all-cause mortality is significantly non-linear across various populations [9, 48]. Specifically, a baseline TyG index below threshold values (< 9.05 for all-cause mortality and < 8.84 for CVD mortality) was negatively associated with mortality, whereas a baseline TyG index above these thresholds showed a positive association with mortality [9].

These findings are consistent with the present study, emphasizing the importance of maintaining an optimal TyG index to reduce health risks. Both extreme too high or too low—may have harmful effects on health.

While these mechanisms provide potential explanations, future studies should explore the interaction between the TyG index, specific medications, and mortality in populations with balanced treatment histories and comorbidities to better clarify this complex relationship.

### Clinical practice

The TyG index, as a simple and readily accessible indicator of IR, has significant potential for broader application in primary care settings. This study demonstrated that antidiabetic and hypolipidemic agents substantially modify the relationship between the TyG index and mortality outcomes. These findings highlight the importance of considering the modifying effects of these agents in future research.

For patients using medications that may influence the TyG index, such as antidiabetic or hypolipidemic agents, different standardized cutoff values may be necessary to improve its predictive ability for cardiovascular events and mortality. This study identified a TyG index cutoff value of approximately 9 as a useful threshold for identifying individuals at higher risk of cardiovascular events and mortality, particularly among older populations. Previous research supports this finding. For example, Wang et al. reported that a TyG index cutoff of 9.323 effectively predicted major adverse cardiovascular events (MACEs) in patients with diabetes and acute coronary syndrome treated with insulin or hypoglycemic medications, enhancing risk stratification models [55]. Similarly, Otsuka et al. identified a TyG index threshold of 8.8 as a reliable predictor of cardiovascular risk in patients with chronic coronary syndrome receiving calcium channel blockers and other medications [56]. Moreover, a TyG index value of 9.45 has been identified as a critical threshold for assessing all-cause mortality in elderly patients, particularly those aged 60 years or older. Studies have shown that exceeding this threshold increases the risk of all-cause mortality by 48% for each standard deviation increase in the TyG index [57]. These findings underscore the need for further research to refine TyG index thresholds, taking into account medication use and other clinical factors, to optimize its predictive value for cardiovascular risk.

### Strengths and limitations

The strengths of this study include its nationwide scope, large sample size, and prospective design, which enabled robust statistical analyses and reliable estimation of the effects of antidiabetic and hypolipidemic agents on the relationship between the TyG index and cardiovascular outcomes. Additionally, sensitivity analyses confirmed the findings, increasing the robustness of the results.

However, this study has several limitations. First, as an observational study, it cannot establish causality. Second, medication use in the NHANES database is based on self-reported data, which is subject to recall bias and potential misclassification. These limitations may lead to inaccuracies in categorizing medication exposure and could affect the reliability of the findings. Furthermore, variability in medication timing, dosage, and types used could significantly influence the outcomes. However, the NHANES dataset lacks information on medication, timing and dosage, which may impact the stability of the results. Third, only baseline TyG index values were analyzed, and future studies should consider assessing the trajectory of the TyG index over time. Fourth, the generalizability of these findings to other regions remains uncertain due to the lack of relevant data in NHANES. Variations in genetic backgrounds, ethnicities, dietary habits, and disease prevalence may influence the applicability of the results. Nonetheless, findings from other studies suggest that these patterns may be applicable to diverse populations [48, 58]. Finally, while the TyG index is a widely used surrogate marker for IR due to its efficiency and accessibility, it remains a subject of ongoing research. Its accuracy, particularly in diverse populations, requires further validation to ensure its reliability across various clinical and epidemiological settings.

## Conclusion

This study showed that the TyG index is significantly correlated with IR measures and is influenced by the use of antidiabetic or hypolipidemic agents. Notably, the use of these agents significantly altered the relationship between the TyG index and mortality outcomes. These findings highlight the importance of considering both TyG index levels and medication use when evaluating mortality risk. Further research is needed to investigate the mechanisms underlying this interaction.

## Electronic supplementary material

Below is the link to the electronic supplementary material.

Supplementary Material 1

## Data Availability

The datasets generated and analyzed in this study were available from the NHANES database, https://www.cdc.gov/nchs/nhanes/.

## Abbreviations

TyG index: Triglyceride Glucose index
BMI: Body mass index
CHD: Coronary heart disease
CI: Confidence Intervals
CVD: Cardiovascular disease
DM: Diabetes mellitus
HDL-C: High-density lipoprotein cholesterol
HF: Heart failure
HOMA-β: Homeostasis model assessment of β-cell function
HOMA-IR: Homeostasis model assessment of insulin resistance
HRs: Hazard ratios
IPTW: Inverse probability treatment weighting
IR: Insulin resistance
LDL-C: Low-density lipoprotein cholesterol
QUICKI: Quantitative insulin sensitivity check index
SE: Standard error
