# Supplementary material for: The association between TyG index and cardiovascular mortality is modified by antidiabetic or lipid-lowering agent: a prospective cohort study
Source: Cardiovasc Diabetol. 2025 Feb 7;24:65. doi: 10.1186/s12933-025-02620-z (PMC11806668; doi:10.1186/s12933-025-02620-z)
Supplement: Supplementary file 1 — Supplementary Material 1 [file 12933_2025_2620_MOESM1_ESM.docx]

**Online Materials**

**The association between TyG index and cardiovascular mortality is modified by antidiabetic or lipid-lowering agent: a prospective cohort study**

Changchang Fang§1, M.D., Nanqin Peng§1, M.D.,Jiang Cheng2, M.D., Xiyu Zhang1, M.D., Wenli Gu3, Ph.D., Zhicheng Zhu2, M.D., Xiaoping Yin4,5, M.D., Zhiwei Yan6, M.D., Jing Zhang2, M.D., Peng Yu1, M.D., Xiao Liu7,8, M.D., Ph.D.

1Department of Endocrinology and Metabolism, the Second Affiliated Hospital, Jiangxi Medical College, Nanchang University, Nanchang, Jiangxi, China

2 Department of Anesthesiology, the Second Affiliated Hospital, Jiangxi Medical College, Nanchang University, Nanchang, Jiangxi, China

3 Cardiology Division, Department of Medicine, The University of Hong Kong, Queen Mary Hospital, Hong Kong, China

4 Department of Neurology, Affiliated Hospital of Jiujiang University, Jiujiang, China

5 Jiujiang Clinical Precision Medicine Research Center, Affiliated Hospital of Jiujiang University, Jiujiang, China

6 Provincial University Key Laboratory of Sport and Health Science, School of Physical Education and Sport Sciences, Fujian Normal University, Fuzhou, Fujian, China.

7 Department of Cardiology, Sun Yat-sen Memorial Hospital of Sun Yat-sen University, Guangzhou, Guangdong, China

8 Cardiovascular & Metabolic Disorders Program, Duke-National University of Singapore Medical School, Singapore, Singapore

§Co-first authors

*Correspondence:

Xiao Liu, Department of Cardiology, Sun Yat-sen Memorial Hospital of Sun Yat-sen University

E-mail: liux587@mail.sysu.edu.cn; kellyclarkwei@vip.qq.com

Peng Yu

E-mail: yu8220182@163.com

Department of Endocrinology and Metabolism, the Second Affiliated Hospital, Jiangxi Medical College, Nanchang University, Nanchang, China

**Table of Contents**

| Table S1 | Baseline characteristics by if used antidiabetic agents of adult Americans in the cohort from the Nation Health and Nutrition Examination Survey 1999-2018. | P4 |
| --- | --- | --- |
| Table S2 | Baseline characteristics by if used hypolipidemic agents of adult Americans in the cohort from the Nation Health and Nutrition Examination Survey 1999-2018 | P6 |
| Table S3 | Baseline characteristics by if used hypolipidemic agents of adult Americans in the cohort from the Nation Health and Nutrition Examination Survey 1999-2018 | P8 |
| Table S4 | The associations of tyg index with all-cause and cardiovascular mortality in the cohort of if used hypolipidemic agents from the Nation Health and Nutrition Examination Survey 1999-2018 after inverse probability treatment weighting. | P10 |
| Table S5 | The associations of TyG index with all-cause and cardiovascular mortality in the cohort of if used hypolipidemic agents from the Nation Health and Nutrition Examination Survey 1999-2018 after inverse probability treatment weighting. | P12 |
| Table S6 | Stratified analyses of the associations of TyG index with all-cause and cardiovascular mortality in the cohort of if used antidiabetic agents from the Nation Health and Nutrition Examination Survey 1999-2018 | P14 |
| Table S7 | Stratified analyses of the associations of TyG index with all-cause and cardiovascular mortality in the cohort of if used hypolipidemic agents from the Nation Health and Nutrition Examination Survey 1999-2018 | P16 |
| Table S8 | Distribution of the breakdown of medication usage among the study population | P18 |
| Figure S1 | Study selection from the NHANES 2011-2016; workflow and major findings of this study. | P19 |
| Figure S2 | Relationship between tyg index and other insulin resistance indices according to both agents use. | P20 |
| Figure S3 | Crude death rates according to subgroup of the tyg index and the antidiabetic agent’s usage. | P21 |
| Figure S4 | Crude death rates according to subgroup of the tyg index and the hypolipemic agents’ usage. | P22 |
| Figure S5 | Weighted Kaplan-Meier analysis of usage of the agent and mortality outcomes according to the agent use. | P23 |
| Figure S6 | Association between TyG index and all-cause and CVD mortality in general population according to both agents. | P24 |
| Figure S7 | Association between TyG index and all-cause and CVD mortality in general population according to both agents, after excluding the population that dead within 2 years. | P25 |
| Figure S8 | Association between TyG index and all-cause and CVD mortality in general population according to antidiabetic agent | P26 |
| Figure S9 | Association between TyG index and all-cause and CVD mortality in general population according to hypolipidemic agent | P27 |
| Figure S10 | Distribution of the TyG index among the study population | P28 |

**Table S1：****Baseline characteristics of adult Americans from the Nation Health and Nutrition Examination Survey 1999-2018**

| **Characteristics** | **Population estimates** | **Observations** |
| --- | --- | --- |
| **Overall** | 42,753,806 | 5,046 |
| TyG index | 8.97(0.02) | 5,046 |
| Age, years | 61.08(0.24) | 5,046 |
| BMI, kg/m2 | 30.93 | 4,953 |
| Waist circumference, cm | 106.29 | 4,807 |
| Female, n (%) | 49.35% | 2,479 |
| **Race, n (%)** |  |  |
| Mexican | 10.41% | 1,016 |
| African American | 5.47% | 763 |
| Caucasian | 73.08% | 2,384 |
| Other Hispanic | 11.04% | 883 |
| **Education, n (%)** |  |  |
| Less than high school | 34.38% | 1,714 |
| High school grad or equivalent | 37.97% | 1,985 |
| Some college or above | 27.44% | 1,331 |
| **Marital status, n (%)** |  |  |
| Married | 63.44% | 2,984 |
| Other | 35.87% | 2,032 |
| **Drinking status, n (%)** |  |  |
| Now | 59.41% | 2,640 |
| Former | 19.90% | 1,153 |
| Never | 11.98% | 7,48 |
| **Smoking status, n (%)** |  |  |
| Now | 16.35% | 792 |
| Former | 36.65% | 1,797 |
| Never | 46.86% | 2,446 |
| **Laboratory results**, mean (SE) |  |  |
| FPG, mg/dL | 127.44(0.96) | 5,046 |
| TC, mg/dL | 187.61(1.06) | 5,046 |
| TG, mg/dL | 155.45(2.40) | 5,046 |
| LDL-C, mg/dL | 105.77(0.88) | 4,896 |
| HDL-C, mg/dL | 51.37(0.31) | 5,046 |
| Scr, umol/L | 120.32(1.28) | 4,951 |
| HbA1C, % | 6.37(0.03) | 5,037 |
| Albumin, g/dL | 4.17(0.01) | 5,027 |
| **Disease**, n (%) |  |  |
| HF | 7.09% | 428 |
| CHD | 12.93% | 629 |
| Angina | 8.44% | 398 |
| Heart attack | 11.42% | 610 |
| Stroke | 8.00% | 452 |
| Hyperlipidemia | 94.77% | 4,704 |
| DM | 65.44% | 3,593 |
| Hypertension | 69.92% | 3,724 |

Note: Data are expressed as mean (SE) and numbers (percentage) as appropriate. All estimates were weighted to be nationally representative.

Abbreviations: BMI: body mass index; TyG index: triglyceride-glucose index; FPG: fasting plasma glucose; TC: total cholesterol; LDL-C: lower-density lipoprotein cholesterol; HDL-C: high-density lipoprotein cholesterol; Scr: serum creatinine; HbA1C: glycated hemoglobin; HF: heart failure; CHD: coronary heart disease; DM: diabetes mellitus.

**Table S2：Baseline characteristics by if used antidiabetic** **agents of adult Americans in the cohort from the Nation Health and Nutrition Examination Survey 1999-2018**

| Characteristics | Overall | If used antidiabetic agents? | |  |  |  |
| --- | --- | --- | --- | --- | --- | --- |
|  |  | No | Yes | P value |  |  |
| **N (%)** | 3906 | 1577(0.40) | 2329(0.60) | < 0.001 |  |  |
| TyG index | 9.04(0.02) | 8.79(0.02) | 9.25(0.03) | < 0.001 |  |  |
| Age, years | 57.05(0.33) | 53.68(0.52) | 59.84(0.37) | < 0.001 |  |  |
| BMI, kg/m^2^ | 32.22(0.17) | 31.38(0.24) | 32.93(0.21) | < 0.001 | |  |
| Waist circumference, cm | 108.44(0.44) | 105.19(0.54) | 111.16(0.54) | < 0.001 |  |  |
| Female, n (%) | 2022(52.87) | 884(57.64) | 1138(48.90) | < 0.001 | | |
| Statin, n (%) | 1614(39.74) | 398(24.20) | 1216(52.64) | < 0.001 | | |
| Insulin, n (%) | 625(14.12) | 0(0) | 625(25.88) | < 0.001 | | |
| **Race, n (%)** |  |  |  | 0.09 | | |
| Mexican | 899(13.40) | 332(11.83) | 567(14.71) |  | | |
| African American | 744(8.77) | 264(8.65) | 480(8.87) |  | | |
| Caucasian | 811(14.87) | 364(16.10) | 447(13.85) |  | | |
| Other Hispanic | 1452(62.96) | 617(63.42) | 835(62.57) |  | | |
| **Education, n (%)** |  |  |  | < 0.001 | | |
| Less than high school | 966(16.97) | 290(12.29) | 676(20.97) |  | | |
| High school grad or equivalent | 861(25.20) | 342(25.16) | 519(25.43) |  | | |
| Some college or above | 2050(57.41) | 928(62.55) | 1122(53.60) |  | | |
| **Marital status, n (%)** |  |  |  | 0.11 | | |
| Married | 2212(59.63) | 852(58.33) | 1360(61.71) |  | | |
| Other | 1664(39.46) | 711(41.67) | 953(38.29) |  | | |
| **Drinking status, n (%)** |  |  |  | < 0.001 | | |
| Now | 1952(55.93) | 896(68.97) | 1056(56.62) |  | | |
| Former | 899(20.30) | 311(18.99) | 588(25.58) |  | | |
| Never | 612(13.64) | 203(12.04) | 409(17.79) |  | | |
| **Smoking status, n (%)** |  |  |  | 0.16 | | |
| Now | 633(16.44) | 284(17.89) | 349(15.29) |  | | |
| Former | 1278(34.17) | 474(32.47) | 804(35.71) |  | | |
| Never | 1978(49.18) | 811(49.64) | 1167(48.99) |  | | |
| **Laboratory results**, mean (SE) |  |  |  |  | | |
| FPG, mg/dL | 139.40(1.38) | 116.15(1.03) | 158.71(1.85) | < 0.001 | | |
| TC, mg/dL | 187.88(1.14) | 196.85(1.58) | 180.44(1.45) | < 0.001 | | |
| TG, mg/dL | 156.94(3.66) | 138.88(4.27) | 171.93(4.92) | < 0.001 | | |
| LDL-C, mg/dL | 108.34(0.91) | 118.67(1.38) | 99.56(1.11) | < 0.001 | | |
| HDL-C, mg/dL | 49.59(0.32) | 51.61(0.50) | 47.91(0.41) | < 0.001 | | |
| HbA1C, % | 6.73(0.04) | 5.91(0.03) | 7.41(0.04) | < 0.001 | | |
| Albumin, g/dL | 4.13(0.01) | 4.17(0.01) | 4.10(0.01) | < 0.001 | | |
| **Disease**, n (%) |  |  |  |  | | |
| HF | 319(6.72) | 83(3.73) | 236(9.26) | < 0.001 | | |
| CHD | 358(8.93) | 105(6.05) | 253(11.47) | < 0.001 | | |
| Angina | 245(6.58) | 74(4.72) | 171(8.24) | 0.002 | | |
| Heart attack | 379 8.85) | 109(6.03) | 270(11.28) | < 0.001 | | |
| Stroke | 304(6.60) | 95(4.68) | 209(8.25) | 0.002 | | |
| Hyperlipidemia | 3323(85.56) | 1250(80.30) | 2073(89.92) | < 0.001 | | |
| DM | 3349(82.74) | 1036(63.48) | 2313(99.61) | < 0.001 | | |
| Hypertension | 2720(66.37) | 974(59.69) | 1746(71.91) | < 0.001 | | |

Note: Data are expressed as mean (SE) and numbers (percentage) as appropriate. All estimates were weighted to be nationally representative.

Abbreviations: BMI: body mass index; TyG index: triglyceride-glucose index; FPG: fasting plasma glucose; TC: total cholesterol; LDL-C: lower-density lipoprotein cholesterol; HDL-C: high-density lipoprotein cholesterol; HF: heart failure; CHD: coronary heart disease; DM: diabetes mellitus.

**Table S3：Baseline characteristics by if used hypolipidemic agents of adult Americans in the cohort from the Nation Health and Nutrition Examination Survey 1999-2018**

| Characteristics | Overall | If used hypolipidemic agents? | |  | |  |  |
| --- | --- | --- | --- | --- | --- | --- | --- |
|  |  | No | Yes | | P value |  |  |
| **N (%)** | 4832 | 1061(0.22) | 3771(0.78) | | < 0.001 |  |  |
| TyG index | 8.92(0.02) | 8.97(0.03) | 8.91(0.02) | | 0.08 |  |  |
| Age, years | 60.78(0.22) | 55.76(0.52) | 62.18(0.24) | | < 0.001 |  |  |
| BMI, kg/m^2^ | 30.34(0.14) | 30.10(0.27) | 30.41(0.16) | | 0.3 | |  |
| Waist circumference, cm | 104.78(0.34) | 103.06(0.64) | 105.25(0.39) | | 0.004 |  |  |
| Female | 2380(49.17) | 545(50.09) | 1835(48.91) | | 0.59 | | |
| Statin, n (%) | 2975(63.20) | 43(3.90) | 2932(79.70) | | < 0.001 | | |
| Insulin, n (%) | 397(6.45) | 33(2.34) | 364(7.70) | | < 0.001 | | |
| **Race, n (%)** |  |  |  | | 0.004 | | |
| Mexican | 877(9.07) | 182(9.40) | 695(8.98) | |  | | |
| African American | 671(5.06) | 179(7.09) | 492(4.49) | |  | | |
| Caucasian | 877(10.68) | 233(12.63) | 644(10.14) | |  | | |
| Other Hispanic | 2407(75.19) | 467(70.88) | 1940(76.39) | |  | | |
| **Education, n (%)** |  |  |  | | 0.03 | | |
| Less than high school | 1028(14.50) | 210(13.77) | 818(14.72) | |  | | |
| High school grad or equivalent | 1180(26.14) | 227(22.54) | 953(27.18) | |  | | |
| Some college or above | 2615(59.24) | 621(63.69) | 1994(58.10) | |  | | |
| **Marital status, n (%)** |  |  |  | | 0.16 | | |
| Married | 2886(63.93) | 609(61.88) | 2277(65.10) | |  | | |
| Other | 1922(35.34) | 444(38.12) | 1478(34.90) | |  | | |
| **Drinking status, n (%)** |  |  |  | | 0.22 | | |
| Now | 2671(61.83) | 634(70.52) | 2037(66.57) | |  | | |
| Former | 1035(19.09) | 196(18.70) | 839(21.40) | |  | | |
| Never | 665(10.79) | 128(10.79) | 537(12.04) | |  | | |
| **Smoking status, n (%)** |  |  |  | | 0.003 | | |
| Now | 1028(14.50) | 217(21.76) | 571(15.96) | |  | | |
| Former | 1180(26.14) | 337(33.34) | 1402(37.65) | |  | | |
| Never | 2615(59.24) | 507(44.91) | 1796(46.39) | |  | | |
| **Laboratory results**, mean (SE) |  |  |  | |  | | |
| FPG, mg/dL | 118.67(0.73) | 113.80(1.37) | 120.03(0.88) | | < 0.001 | | |
| TG, mg/dL | 159.96(2.82) | 177.97(7.35) | 154.95(2.68) | | 0.002 | | |
| LDL-C, mg/dL | 113.05(0.94) | 148.13(1.70) | 103.56(0.94) | | < 0.001 | | |
| HDL-C, mg/dL | 52.02(0.32) | 51.33(0.72) | 52.21(0.36) | | 0.29 | | |
| HbA1C, % | 6.08(0.02) | 5.86(0.04) | 6.14(0.03) | | < 0.001 | | |
| Albumin, g/dL | 4.20(0.01) | 4.20(0.02) | 4.20(0.01) | | 0.93 | | |
| **Disease**, n (%) |  |  |  | |  | | |
| HF | 382(6.56) | 46(3.43) | 336(7.47) | | < 0.001 | | |
| CHD | 606(12.72) | 60(5.09) | 546(14.95) | | < 0.001 | | |
| Angina | 372(8.31) | 46(4.96) | 326(9.29) | | 0.002 | | |
| Heart attack | 567(11.00) | 69(5.34) | 498(12.59) | | < 0.001 | | |
| Stroke | 402(7.37) | 49(3.34) | 353(8.50) | | < 0.001 | | |
| Hyperlipidemia | 4632(97.12) | 949(91.85) | 3683(98.58) | | < 0.001 | | |
| DM | 2907(54.73) | 517(44.26) | 2390(57.71) | | < 0.001 | | |
| Hypertension | 3506(68.43) | 653(58.92) | 2853(71.07) | | < 0.001 | | |

Note: Data are expressed as mean (SE) and numbers (percentage) as appropriate. All estimates were weighted to be nationally representative.

Abbreviations: BMI: body mass index; TyG index: triglyceride-glucose index; FPG: fasting plasma glucose; TC: total cholesterol; LDL-C: lower-density lipoprotein cholesterol; HDL-C: high-density lipoprotein cholesterol; HF: heart failure; CHD: coronary heart disease; DM: diabetes mellitus.

**Table S4: The associations of TyG index with all-cause and cardiovascular mortality in the cohort of if used antidiabetic** **agents from the Nation Health and Nutrition Examination Survey 1999-2018 after inverse probability treatment weighting.**

|  | | **Number of deaths (death rate)** | | **Crude Model**  **HR (95%CI)** | **P** | **Model I**  **HR (95%CI)** | **P** | **Model II**  **HR (95%CI)** | **P** | **P for interaction** |
| --- | --- | --- | --- | --- | --- | --- | --- | --- | --- | --- |
| **All-cause mortality** | | | | | | | | | | |
| **Without antidiabetic agent** | | | | | | | | | | |
| TyG index | | | | | | | | | | |
| T1 (≤8.69) | | 73(10.75) | | Ref |  | Ref |  | Ref |  | 0.001 |
| T2 (8.69-9.27) | | 79(10.78) | | 0.90(0.35,2.30) | 0.82 | 1.03(0.56,1.92) | 0.92 | 1.89(1.01,3.55) | 0.05 |  |
| T3 (≥9.27) | | 61(7.63) | | 0.54(0.20,1.46) | 0.22 | 0.48(0.23,1.00) | 0.05 | 1.57(0.87,2.85) | 0.14 |  |
| P for trend | |  |  |  | 0.28 |  | 0.06 |  | 0.09 |  |
| **With antidiabetic agent** | | | | | | | | | | |
| TyG index |  | | | | | | | | | |
| T1 (≤8.69) | | 143(25.48) | | 1.35(1.07,1.70) | 0.01 | 1.36(1.07,1.73) | 0.01 | 1.23(0.95,1.60) | 0.12 |  |
| T2 (8.69-9.27) | | 186(22.63) | | Ref |  | Ref |  | Ref |  |  |
| T3 (≥9.27) | | 284(27.32) | | 1.13(0.90,1.42) | 0.30 | 1.34(1.09,1.66) | 0.01 | 1.19(0.92,1.56) | 0.19 |  |
| P for trend | |  | |  | 0.33 |  | 0.75 |  | 0.98 |  |
| **CVD mortality** | | | | | | | | | | |
| **Without antidiabetic agent** | | | | | | | | | | |
| TyG index |  | | | | | | | | | |
| T1 (≤8.69) | | 18(0.85) | | Ref |  | Ref |  | Ref |  | 0.04 |
| T2 (8.69-9.27) | | 21(3.85) | | 4.07(1.55,10.70) | 0.004 | 3.79(1.31,11.00) | 0.01 | 3.68(1.08,12.49) | 0.04 |  |
| T3 (≥9.27) | | 15(1.66) | | 1.45(0.54, 3.87) | 0.46 | 1.08(0.33, 3.58) | 0.90 | 1.62(0.38, 6.83) | 0.51 |  |
| P for trend | |  | |  | 0.26 |  | 0.72 |  | 0.46 |  |
| **With antidiabetic agent** | | | | | | | | | | |
| TyG index | | | | | | | | | | |
| T1 (≤8.69) | | 49(9.87) | | 1.85(1.15,2.95) | 0.01 | 1.95(1.21,3.14) | 0.01 | 2.06(1.26,3.37) | 0.004 |  |
| T2 (8.69-9.27) | | 54(6.48) | | Ref |  | Ref |  | Ref |  |  |
| T3 (≥9.27) | | 89(7.48) | | 1.06(0.70,1.62) | 0.77 | 1.23(0.82,1.83) | 0.32 | 1.14(0.71,1.84) | 0.59 |  |
| P for trend | |  |  |  | 0.03 |  | 0.11 |  | 0.04 |  |

Note: IPTW adjusted age, sex, race, educational status, BMI, marital status, waist circumference, alcohol using status, smoking status, FPG, TC, triglyceride, LDL-C, HDL-C, HbA1C, CHD, angina, heart attack, stroke, HF, hyperlipidemia, hypertension

Model I: Adjusted for age, sex, race.

Model II: Adjusted for age, sex, race, BMI, educational status, hypertension, CHD, stroke, angina

Abbreviations: 95% CI: 95% confidence interval; HR: hazard ratio; Ref: reference; BMI: body mass index; TyG: triglyceride-glucose index; CHD: coronary heart disease.

**Table S5: The associations of TyG index with all-cause and cardiovascular mortality in the cohort of if used hypolipidemic agents from the Nation Health and Nutrition Examination Survey 1999-2018 after inverse probability treatment weighting.**

|  | | **Number of deaths (death rate)** | | | **Crude Model**  **HR (95%CI)** | **P** | **Model I**  **HR (95%CI)** | **P** | **Model II**  **HR (95%CI)** | **P** | **P for interaction** |
| --- | --- | --- | --- | --- | --- | --- | --- | --- | --- | --- | --- |
| **All-cause mortality** | | | | | | | | | | | |
| **Without hypolipidemic agent** | | | | | | | | | | | |
| TyG index | | | | | | | | | | | |
| T1 (≤8.59) | | 42(14.64) | | | Ref |  | Ref |  | Ref |  | 0.03 |
| T2 (8.59-9.14) | | 60(16.64) | | | 1.01(0.49,2.05) | 0.99 | 0.88(0.40,1.90) | 0.74 | 1.00(0.43,2.30) | 1.00 |  |
| T3 (≥9.14) | | 66(13.24) | | | 0.82(0.44,1.53) | 0.53 | 1.23(0.64,2.34) | 0.53 | 1.12(0.49,2.55) | 0.79 |  |
| P for trend | |  |  |  | | 0.45 |  | 0.27 |  | 0.73 |  |
| **With hypolipidemic agent** | | | | | | | | | | | |
| TyG index |  | | | | | | | | | | |
| T1 (≤8.59) | | 274(18.82) | | | 1.10(0.65,1.86) | 0.73 | 1.16(0.75,1.80) | 0.51 | 1.21(0.85,1.73) | 0.29 |  |
| T2 (8.59-9.14) | | 286(15.25) | | | Ref |  | Ref |  | Ref |  |  |
| T3 (≥9.14) | | 314(20.14) | | | 1.06(0.70,1.59) | 0.79 | 1.69(1.13,2.53) | 0.01 | 1.47(1.07,2.04) | 0.02 |  |
| P for trend | |  | | |  | 0.73 |  | 0.46 |  | 0.24 |  |
| **CVD mortality** | | | | | | | | | | | |
| **Without hypolipidemic agent** | | | | | | | | | | | |
| TyG index |  | | | | | | | | | | |
| T1 (≤8.59) | | 10(4.69) | | | Ref |  | Ref |  | Ref |  | 0.04 |
| T2 (8.59-9.14) | | 18(8.22) | | | 1.56(0.41,5.94) | 0.52 | 1.17(0.26, 5.25) | 0.83 | 1.58(0.34, 7.31) | 0.56 |  |
| T3 (≥9.14) | | 14(3.71) | | | 0.67(0.18,2.59) | 0.56 | 1.23(0.31, 4.81) | 0.77 | 1.09(0.21, 5.60) | 0.92 |  |
| P for trend | |  | | |  | 0.11 |  | 0.98 |  | 0.5 |  |
| **With hypolipidemic agent** | | | | | | | | | | | |
| TyG index | | | | | | | | | | | |
| T1 (≤8.59) | | 89(6.49) | | | 1.21(0.66,2.22) | 0.55 | 1.22(0.71,2.09) | 0.47 | 1.34(0.85,2.13) | 0.21 |  |
| T2 (8.59-9.14) | | 84(4.90) | | | Ref |  | Ref |  | Ref |  |  |
| T3 (≥9.14) | | 103(5.98) | | | 0.98(0.58,1.64) | 0.94 | 1.61(0.96,2.69) | 0.07 | 1.42(0.91,2.20) | 0.12 |  |
| P for trend | |  |  |  | | 0.54 |  | 0.44 |  | 0.19 |  |

Note: IPTW adjusted age, sex, race, educational status, marital status, hypertension status, alcohol using status, smoking status, CHD, angina, heart attack, stroke, UA, BMI, TC, LDL-C, HDL-C, Scr

Model I: Adjusted for age, sex, race.

Model II: Adjusted for age, sex, race, BMI, educational status, hypertension, CHD, stroke, angina

Abbreviations: 95% CI: 95% confidence interval; HR: hazard ratio; Ref: reference; BMI: body mass index; TyG: triglyceride-glucose index; CHD: coronary heart disease.

**Table S6 Stratified analyses of the associations of TyG index with all-cause and cardiovascular mortality in the cohort of if used antidiabetic** **agents from the Nation Health and Nutrition Examination Survey 1999-2018.**

|  | **Adjusted Model HR (95%CI)** | | | **P for interaction** | **Adjusted Model HR (95%CI)** | | | **P for interaction** |
| --- | --- | --- | --- | --- | --- | --- | --- | --- |
| **All-cause mortality** | | | | | | | | |
|  | **With antidiabetic agent** | | |  | **Without antidiabetic agent** | | |  |
|  | T1  (≤8.69) | T2  (8.69-9.27) | T3  (≥9.27) |  | T1  (≤8.69) | T2  (8.69-9.27) | T3  (≥9.27) |  |
| **Age** |  |  |  | 0.12 |  |  |  | 0.74 |
| <60 | 1.38(0.61,3.11) | Ref | 1.63(1.17,2.28) |  | Ref | 1.23(0.46,3.29) | 1.74(0.63,4.80) |  |
| ≥60 | 1.33(1.02,1.74) | Ref | 2.23(1.15,4.32) |  | Ref | 1.49(1.00,2.23) | 1.40(0.89,2.20) |  |
| **Sex** |  |  |  | 0.52 |  |  |  | 0.82 |
| Female | 1.16(0.80,1.68) | Ref | 1.63(1.17,2.28) |  | Ref | 1.33(0.79,2.25) | 1.24(0.68,2.29) |  |
| Male | 1.42(1.00,2.01) | Ref | 1.20(0.88,1.64) |  | Ref | 1.45(0.91,2.29) | 1.69(0.92,3.12) |  |
| **BMI** |  |  |  | 0.35 |  |  |  | 0.89 |
| <30 | 1.21(0.87,1.67) | Ref | 1.45(1.04,2.01) |  | Ref | 1.64(1.05,2.58) | 1.82(1.08,3.07) |  |
| ≥30 | 1.44(0.98,2.11) | Ref | 1.14(0.83,1.57) |  | Ref | 1.28(0.57,2.88) | 1.44(0.63,3.26) |  |
| **CVD** |  |  |  | 0.48 |  |  |  | 0.41 |
| Yes | 1.79(1.20,2.67) | Ref | 1.39(0.96,2.01) |  | Ref | 1.76(1.02,3.04) | 1.36(0.69,2.68) |  |
| No | 1.13(0.76,1.67) | Ref | 1.27(0.93,1.75) |  | Ref | 1.17(0.77,1.80) | 1.50(0.83,2.71) |  |
| **CVD mortality** | | | | | | | | |
|  | **With antidiabetic agent** | | | **P for interaction** | **Without antidiabetic agent** | | | **P for interaction** |
|  | T1  (≤8.69) | T2  (8.69-9.27) | T3  (≥9.27) |  | T1  (≤8.69) | T2  (8.69-9.27) | T3  (≥9.27) |  |
| **Age** |  |  |  | 0.19 |  |  |  | 0.05 |
| <60 | 1.82(0.44,7.50) | Ref | 3.46(1.24,9.69) |  | Ref | 1.26(0.48,3.29) | 3.70(0.63,4.02) |  |
| ≥60 | 1.49(0.86,2.56) | Ref | 1.17(0.75,1.81) |  | Ref | 1.06(0.51,2.23) | 0.77(0.31,1.90) |  |
| **Sex** |  |  |  | 0.94 |  |  |  | 0.17 |
| Female | 1.60(0.84,3.05) | Ref | 1.42(0.90,2.23) |  | Ref | 3.00(1.09, 8.29) | 3.99(1.09,14.61) |  |
| Male | 1.29(0.57,2.93) | Ref | 1.74(0.89,3.41) |  | Ref | 1.03(0.40,2.64) | 0.47(0.12,1.81) |  |
| **BMI** |  |  |  | 0.89 |  |  |  | 0.04 |
| <30 | 1.44(0.67,3.09) | Ref | 1.33(0.75,2.36) |  | Ref | 1.44(0.54,3.84) | 0.49(0.15,1.67) |  |
| ≥30 | 1.57(0.82,3.01) | Ref | 1.58(0.92,2.72) |  | Ref | 2.79(0.99, 7.87) | 3.70(0.91,15.02) |  |
| **CVD** |  |  |  | 0.77 |  |  |  | 0.26 |
| Yes | 1.49(0.76,2.92) | Ref | 1.49(0.80,2.75) |  | Ref | 2.17(0.82,5.74) | 0.84(0.28,2.54) |  |
| No | 1.70(0.83,3.50) | Ref | 1.50(0.85,2.67) |  | Ref | 1.11(0.39,3.18) | 1.96(0.44,8.75) |  |

Note: The adjusted model accounts for sex and race in the age subgroup, age and race in the sex subgroup, and age, sex, and race in all other subgroups.

Abbreviations: 95% CI: 95% confidence interval; CVD: cardiovascular disease; HR: hazard ratio; Ref: reference; TyG: triglyceride-glucose index.

**Table S7 Stratified analyses of the associations of TyG index with all-cause and cardiovascular mortality in the cohort of if used hypolipidemic agents from the Nation Health and Nutrition Examination Survey 1999-2018.**

|  | **Adjusted Model HR (95%CI)** | | | **P for interaction** | **Adjusted Model HR (95%CI)** | | | **P for interaction** |
| --- | --- | --- | --- | --- | --- | --- | --- | --- |
| **All-cause mortality** | | | | | | | | |
|  | **With hypolipidemic** **agent** | | |  | **Without hypolipidemic** **agent** | | |  |
|  | T1  (≤8.59) | T2  (8.59-9.14) | T3  (≥9.14) |  | T1  (≤8.59) | T2  (8.59-9.14) | T3  (≥9.27) |  |
| **Age** |  |  |  | 0.98 |  |  |  | 0.74 |
| <60 | 1.33(0.41,4.35) | Ref | 1.39(0.64,3.02) |  | Ref | 1.33(0.41,4.35) | 1.74(0.63,4.80) |  |
| ≥60 | 1.58(1.04,2.41) | Ref | 1.37(0.90,2.09) |  | Ref | 1.58(1.04,2.41) | 1.40(0.89,2.20) |  |
| **Sex** |  |  |  | 0.27 |  |  |  | 0.82 |
| Female | 1.60(0.81,3.15) | Ref | 2.13(1.26,3.58) |  | Ref | 1.60(0.81,3.15) | 1.24(0.68,2.29) |  |
| Male | 1.49(0.83,2.69) | Ref | 1.04(0.64,1.68) |  | Ref | 1.49(0.83,2.69) | 1.69(0.92,3.12) |  |
| **BMI** |  |  |  | 0.95 |  |  |  | 0.89 |
| <30 | 1.38(0.79,2.42) | Ref | 1.22(0.71,2.12) |  | Ref | 1.38(0.79,2.42) | 1.82(1.08,3.07) |  |
| ≥30 | 1.85(1.03,3.34) | Ref | 1.33(0.81,2.18) |  | Ref | 1.85(1.03,3.34) | 1.44(0.63,3.26) |  |
| **CVD** |  |  |  | 0.07 |  |  |  | 0.41 |
| Yes | 3.26(1.75,6.07) | Ref | 1.66(1.01,2.72) |  | Ref | 3.26(1.75,6.07) | 1.36(0.69,2.68) |  |
| No | 0.85(0.47,1.54) | Ref | 1.12(0.66,1.90) |  | Ref | 0.85(0.47,1.54) | 1.50(0.83,2.71) |  |
| **CVD mortality** | | | | | | | | |
|  | **With hypolipidemic** **agent** | | | **P for interaction** | **Without hypolipidemic** **agent** | | | **P for interaction** |
|  | T1  (≤8.69) | T2  (8.69-9.27) | T3  (≥9.27) |  | T1  (≤8.69) | T2  (8.69-9.27) | T3  (≥9.27) |  |
| **Age** |  |  |  | 0.78 |  |  |  | 0.07 |
| <60 | 2.14(0.43,10.77) | Ref | 1.89(0.58, 6.17) |  | Ref | 1.33(0.41,4.35) | 3.70(0.63,4.02) |  |
| ≥60 | 1.07(0.46,2.46) | Ref | 1.11(0.56,2.19) |  | Ref | 1.58(1.04,2.41) | 0.77(0.31,1.90) |  |
| **Sex** |  |  |  | 0.43 |  |  |  | 0.17 |
| Female | 0.69(0.20,2.45) | Ref | 1.63(0.63,4.23) |  | Ref | 1.60(0.81,3.15) | 3.99(1.09,14.61) |  |
| Male | 1.53(0.56,4.18) | Ref | 1.01(0.47,2.17) |  | Ref | 1.49(0.83,2.69) | 0.47(0.12,1.81) |  |
| **BMI** |  |  |  | 0.40 |  |  |  | 0.04 |
| <30 | 1.83(0.61,5.47) | Ref | 1.36(0.57,3.24) |  | Ref | 1.38(0.79,2.42) | 0.49(0.15,1.67) |  |
| ≥30 | 1.77(0.31,1.86) | Ref | 1.02(0.47,2.19) |  | Ref | 1.85(1.03,3.34) | 3.70(0.91,15.02) |  |
| **CVD** |  |  |  | 0.41 |  |  |  | 0.26 |
| Yes | 2.12(0.68,6.60) | Ref | 1.52(0.71,3.24) |  | Ref | 3.26(1.75,6.07) | 0.84(0.28,2.54) |  |
| No | 0.85(0.29,2.52) | Ref | 0.92(0.38,2.21) |  | Ref | 0.85(0.47,1.54) | 1.96(0.44,8.75) |  |

Note: The adjusted model accounts for sex and race in the age subgroup, age and race in the sex subgroup, and age, sex, and race in all other subgroups.

Abbreviations: 95% CI: 95% confidence interval; CVD: cardiovascular disease; HR: hazard ratio; Ref: reference; TyG: triglyceride-glucose index.

**Table S8** **Distribution of the breakdown of medication usage among the study population**

| **Characteristics** | **Population estimates** | **Observations** |
| --- | --- | --- |
| **Overall** | 42,753,806 | 5,046 |
| Antidiabetic agents | | |
| Insulin, n (%) | 10.20% | 625 |
| Metformin, n (%) | 15.84% | 884 |
| Sulfonylureas, n (%) | 4.18% | 272 |
| Thiazolidinediones, n (%) | 3.35% | 170 |
| Dipeptidyl Peptidase-4 Inhibitors, n (%) | 1.94% | 106 |
| Hypolipidemic agent | | |
| Statin, n (%) | 65.87% | 3,165 |
| Simvastatin, n (%) | 22.22% | 1,121 |
| Atorvastatin, n (%) | 21.36% | 1,078 |
| Rosuvastatin, n (%) | 5.09% | 257 |
| Lovastatin, n (%) | 4.66% | 235 |
| Pravastatin, n (%) | 6.52% | 329 |
| Fibrates, n (%) | 5.28% | 234 |
| Bile Acid Sequestrants, n (%) | 0.78% | 32 |
| Other Lipid-Lowering Drugs, n (%) | 5.31% | 230 |

Note: Data are expressed as mean (SE) and numbers (percentage) as appropriate. All estimates were weighted to be nationally representative.

**Figure S1 Study selection from the NHANES 2011-2016; workflow and major findings of this study.**

**
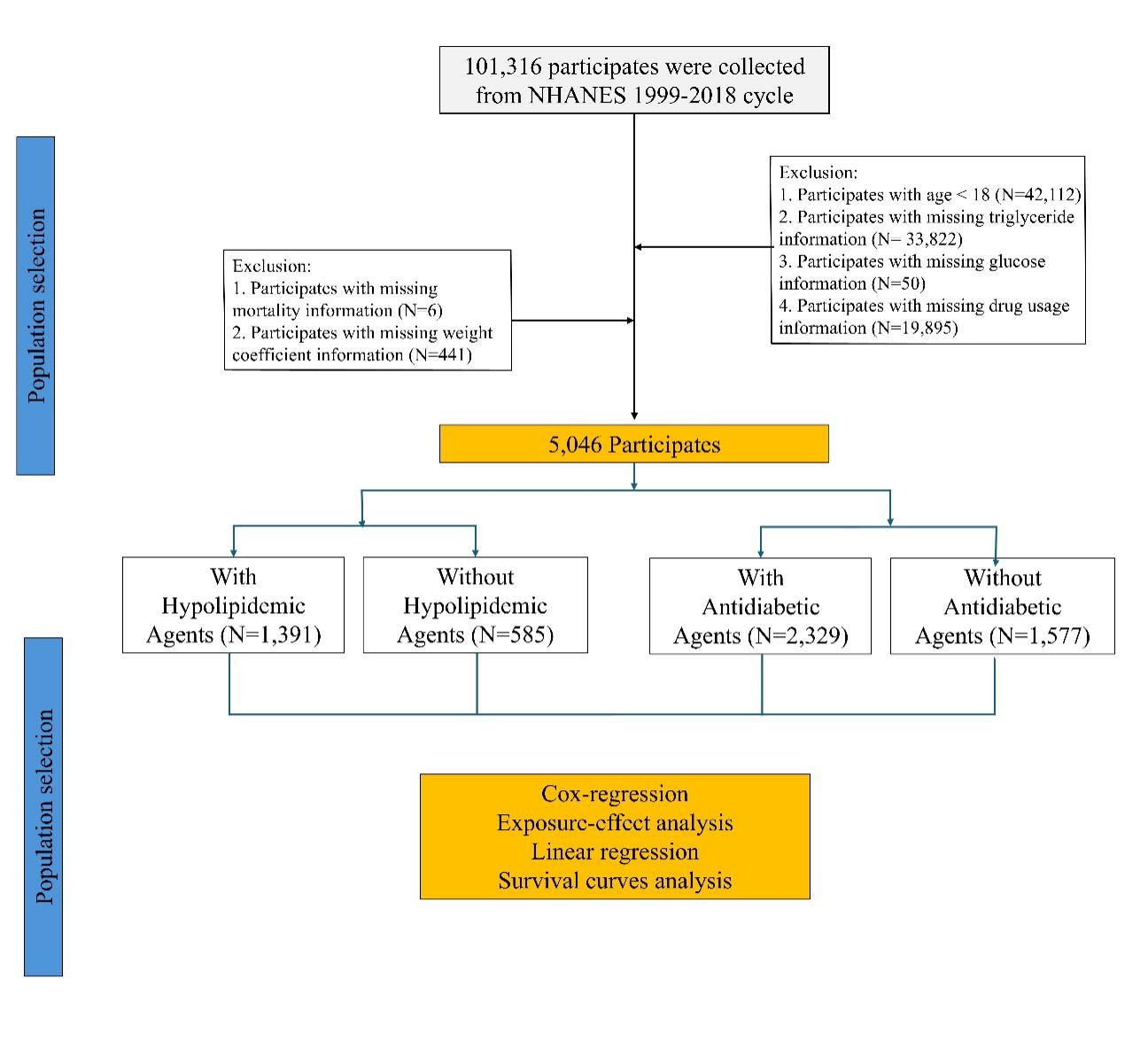
**

**Figure S2 Relationship between TyG index and other insulin resistance indices according to both agents use.**


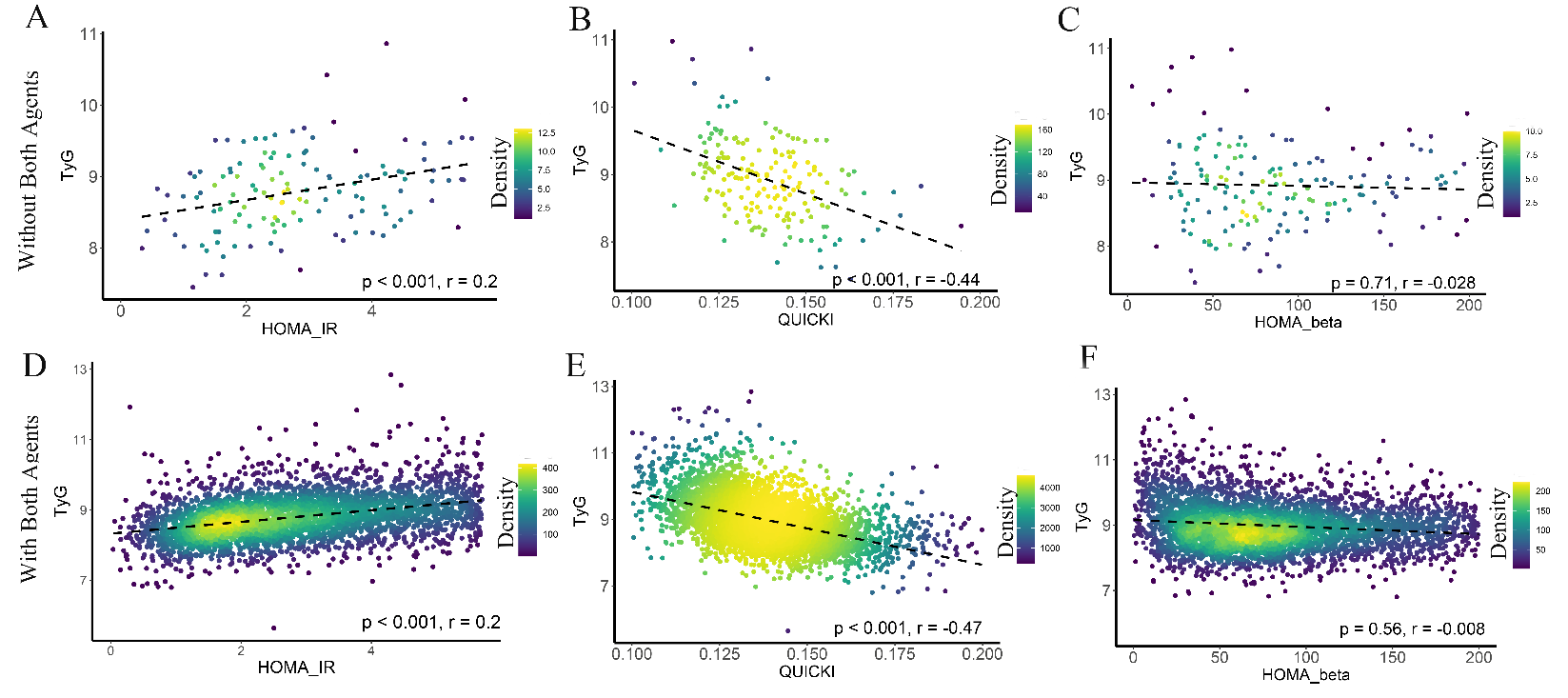


Note: Relationship between the TyG index and other insulin resistance indices (HOMA-IR, QUICKI, HOMA-β), stratified by both use of antidiabetic and hypolipidemic agents by linear regression to analyses the after adjusting for age, sex, and race.

Abbreviations: HOMA-β, homeostasis model assessment of β-cell function; HOMA-IR, homeostasis model assessment of insulin resistance; QUICKI, quantitative insulin sensitivity check index.

**Figure S3. Crude death rates according to subgroup of the TyG index and the antidiabetic** **agent’s usage.**

**
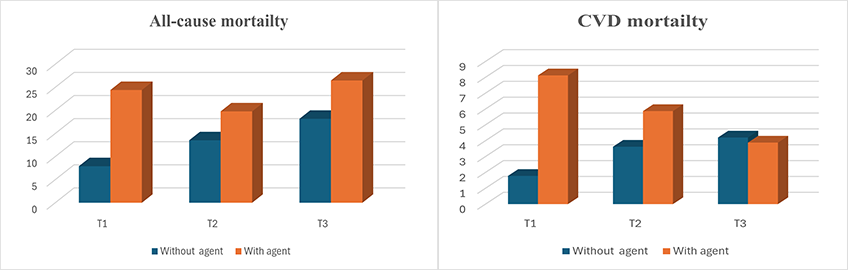
**

**Figure S4 Crude death rates according to subgroup of the TyG index and the hypolipemic agents’ usage.**

**
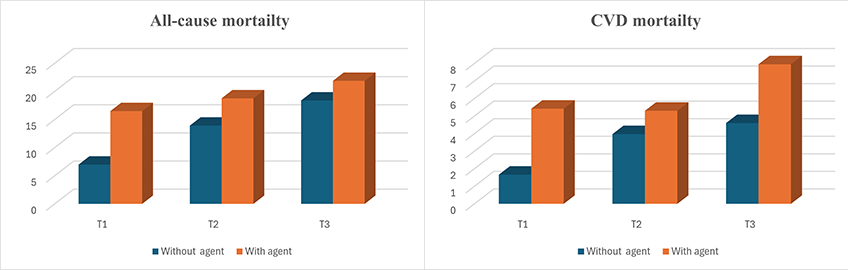
**

**Figure S5 Weighted Kaplan-Meier analysis of usage of the agent and mortality outcomes according to the agent use.**


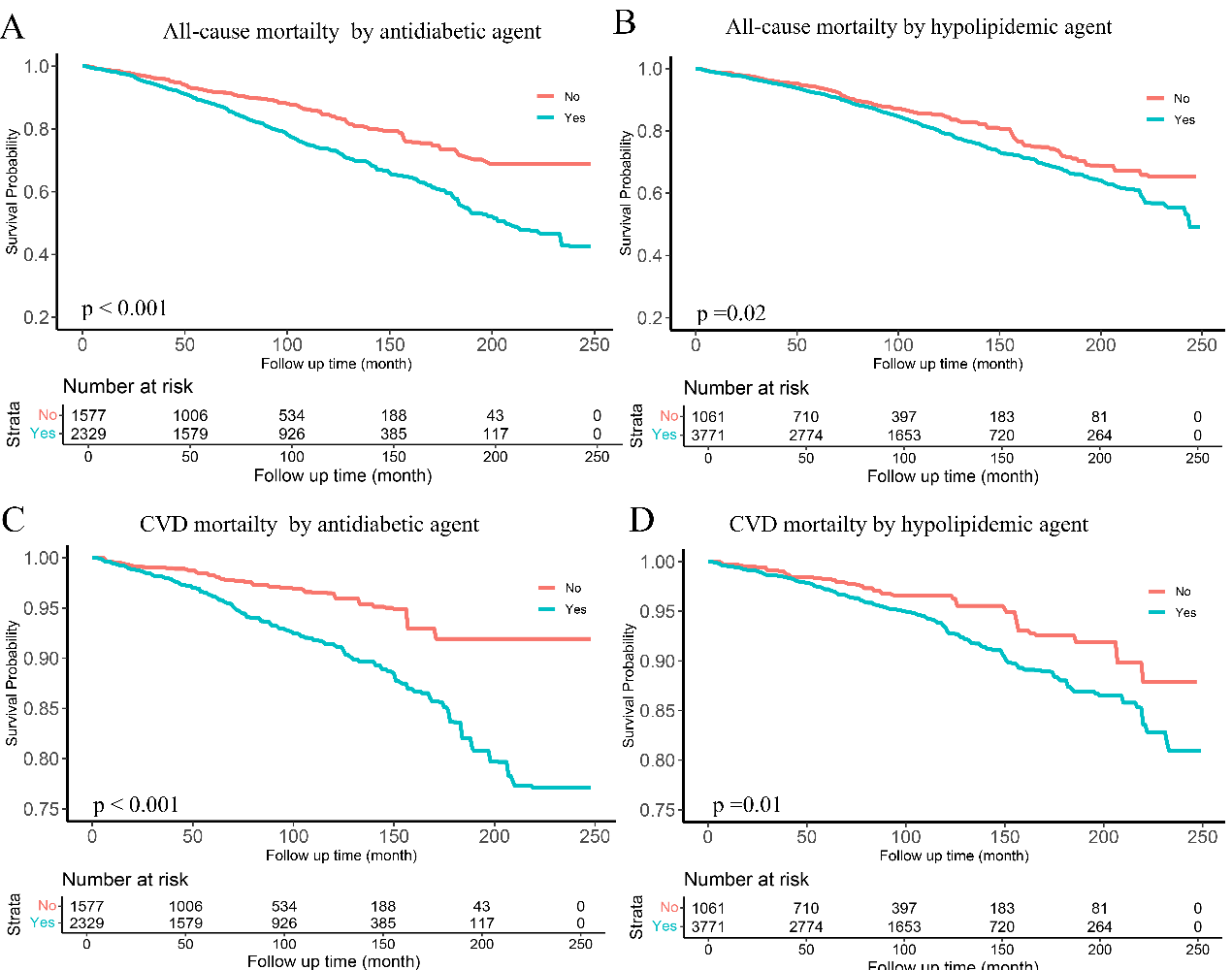


Note: A: Antidiabetic agent usage and all-cause mortality. B: Antidiabetic usage and CVD mortality. C: Hypolipidemic agent usage and all-cause mortality. D: Hypolipidemic agent usage and CVD mortality.

Abbreviation: CVD: cardiovascular disease.

**Figure S6 Association between TyG index and all-cause and CVD mortality in general population according to both agents.**


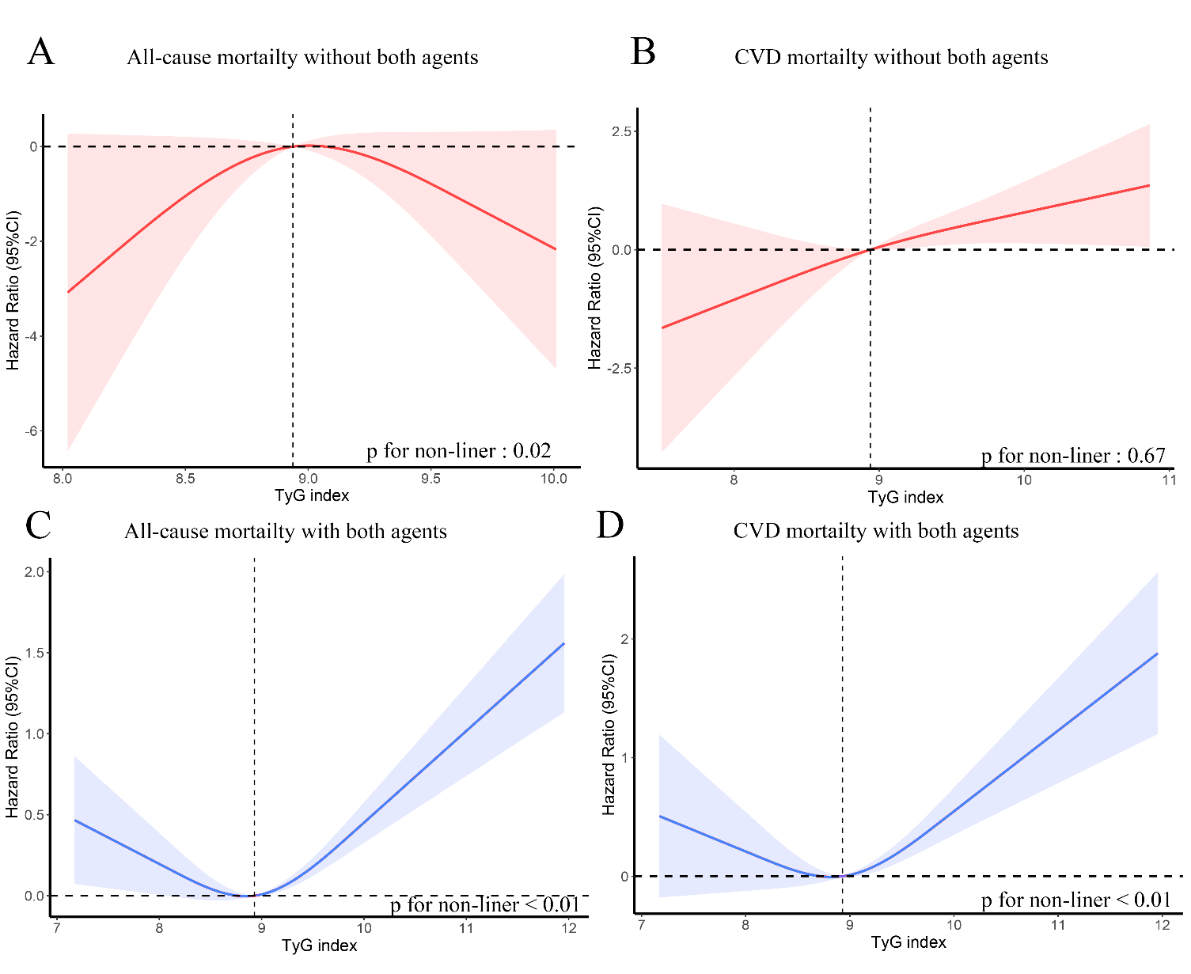


Note: Weighted Cox proportional hazards regression model of TyG index and all-cause (A, C) and CVD mortality (B, D) in general population according to both use of antidiabetic and hypolipidemic agent after adjusting for age, sex, and race.

The hazard ratio of the probability distribution for mortality according to TyG index tertiles. The solid line and area represent the estimated values and their corresponding 95% CIs, respectively.

Abbreviations: CVD: cardiovascular disease; TyG index: triglyceride-glucose index.

**Figure S7 Association between TyG index and all-cause and CVD mortality in general population according to both agents, after excluding the population that dead within 2 years.**


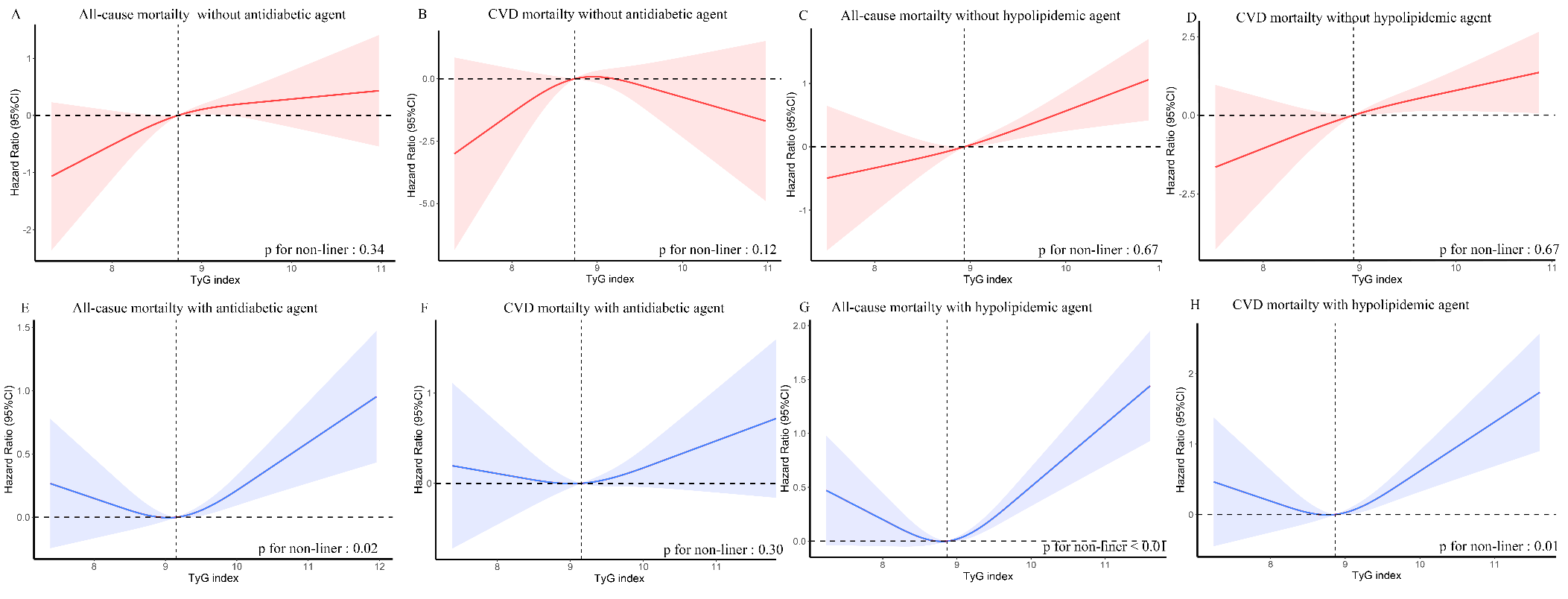


Note: Weighted Cox proportional hazards regression model of TyG index and all-cause (A, C, E, G) and CVD mortality (B, D, F, H) in general population according to both use of antidiabetic and hypolipidemic agent after adjusting for age, sex, race, BMI, hypertension status, CHD status, stroke status, angina status, alcohol using status and smoking status ,after excluding the population that dead within 2 years.

The hazard ratio of the probability distribution for mortality according to TyG index tertiles. The solid line and area represent the estimated values and their corresponding 95% CIs, respectively.

Abbreviations: BMI: body mass index; CHD: coronary heart disease; CVD: cardiovascular disease; TyG index: triglyceride-glucose index.

**Figure S8 Association between TyG index and all-cause and CVD mortality in general population according to antidiabetic agent**


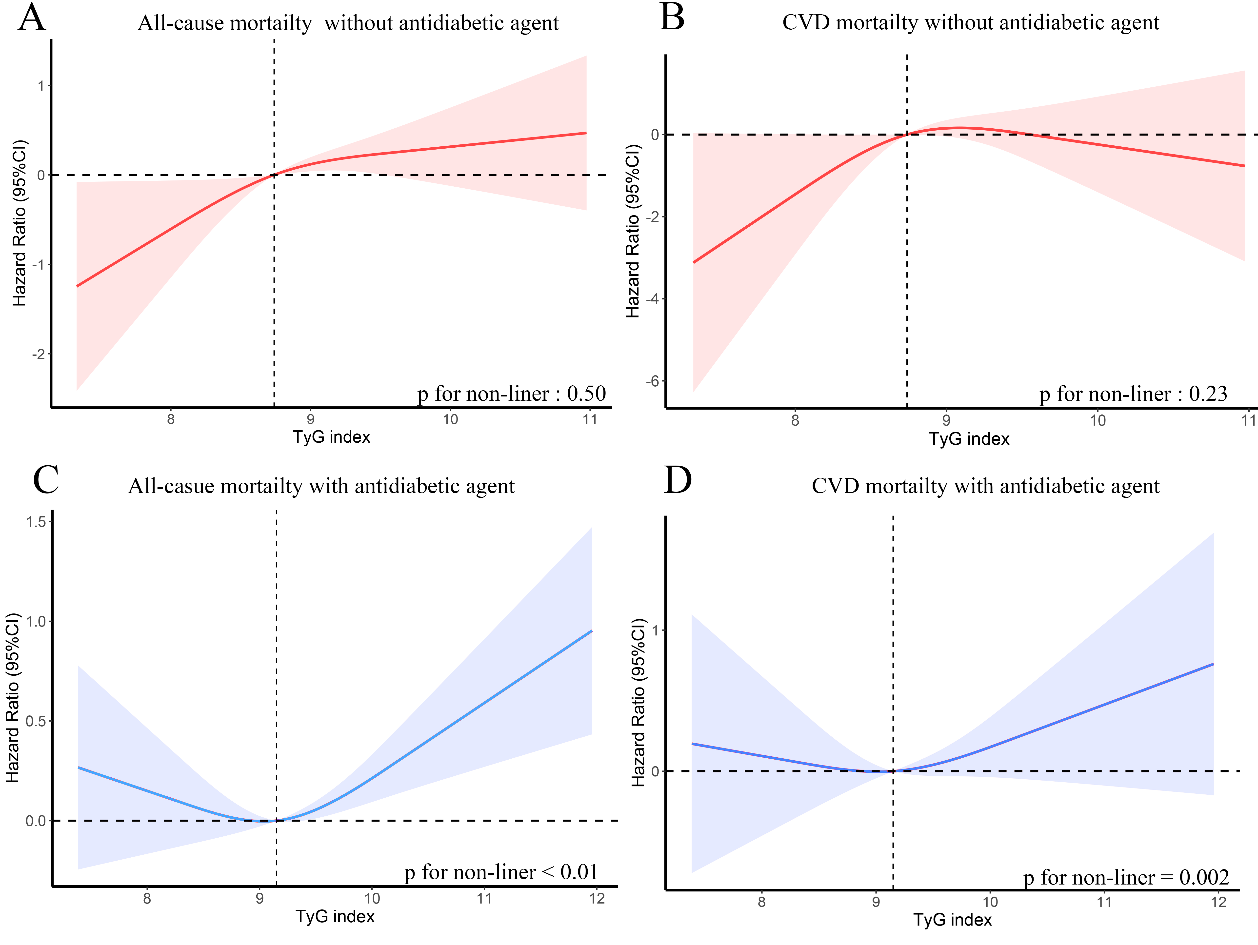


Note: Weighted Cox proportional hazards regression model of TyG index and all-cause (A, C) and CVD mortality (B, D) in general population according to antidiabetic agent after adjusting for age, sex, race, BMI, hypertension status, HEI, physical activity, PIR, alcohol using status and smoking status

The Hazard ratio of the probability distribution for mortality according to TyG index tertiles. The solid line and red area represent the estimated values and their corresponding 95% CIs, respectively.

Abbreviations: BMI: body mass index; CHD: coronary heart disease; CVD: cardiovascular disease; TyG index: triglyceride-glucose index.

**Figure S9 Association between TyG index and all-cause and CVD mortality in general population according to hypolipidemic agent**


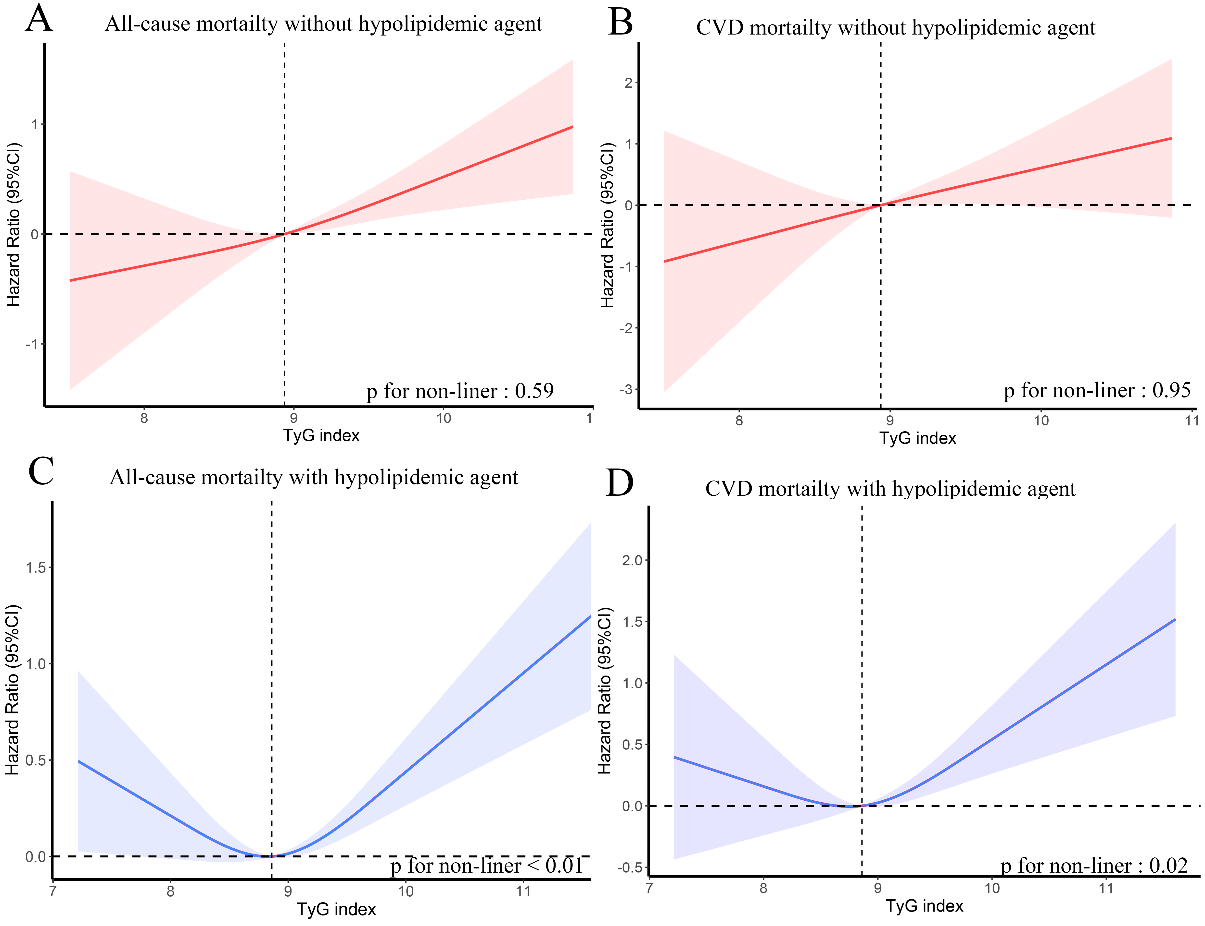


Note: Weighted Cox proportional hazards regression model of TyG index and all-cause (A, C) and CVD mortality (B, D) in general population according to antidiabetic agent after adjusting for age, sex, race, BMI, hypertension status, HEI, physical activity, PIR, alcohol using status and smoking status.

The Hazard ratio of the probability distribution for mortality according to TyG index tertiles. The solid line and red area represent the estimated values and their corresponding 95% CIs, respectively.

Abbreviations: BMI: body mass index; CHD: coronary heart disease; CVD: cardiovascular disease; TyG index: triglyceride-glucose index.

**Figure S10 Distribution of the TyG index among the study population**


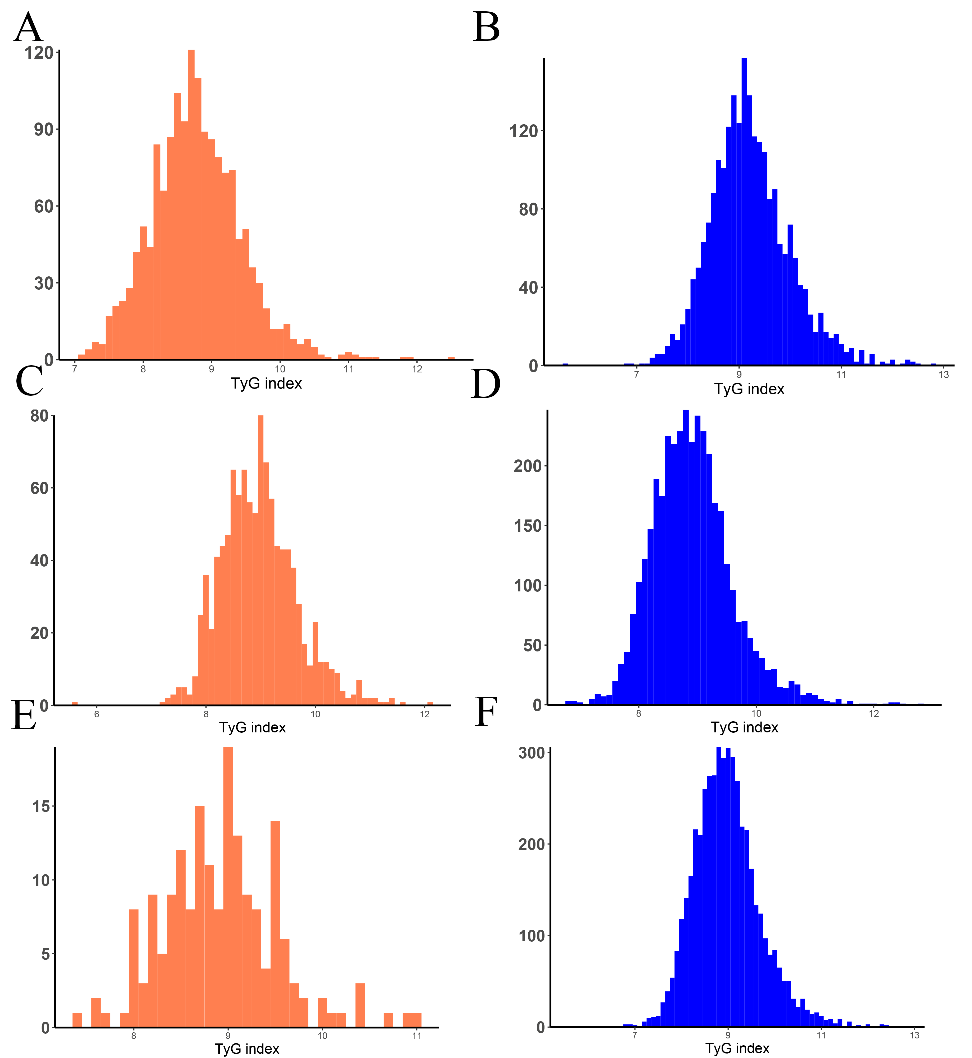


Note: The distribution of the TyG index among the study population is presented as follows: (A) without antidiabetic agent, (B) with antidiabetic agent, (C) without hypolipidemic agent, (D) with hypolipidemic agent, and (E) without both antidiabetic and hypolipidemic agents, and (F) with both agents.
